# Neuroactive Steroids, Toll-like Receptors, and Neuroimmune Regulation: Insights into Their Impact on Neuropsychiatric Disorders

**DOI:** 10.3390/life14050582

**Published:** 2024-04-30

**Authors:** Irina Balan, Giorgia Boero, Samantha Lucenell Chéry, Minna H. McFarland, Alejandro G. Lopez, A. Leslie Morrow

**Affiliations:** 1 Bowles Center for Alcohol Studies, School of Medicine, The University of North Carolina at Chapel Hill, Chapel Hill, NC 27599, USA; iirina@email.unc.edu (I.B.); chery@email.unc.edu (S.L.C.); minna@unc.edu (M.H.M.); alopezsf@live.unc.edu (A.G.L.); 2 Department of Psychiatry, School of Medicine, The University of North Carolina at Chapel Hill, Chapel Hill, NC 27599, USA; 3 Department of Psychiatry and Behavioral Sciences, Duke University Medical Center, Durham, NC 27710, USA; giorgia.boero@gmail.com; 4 Neuroscience Curriculum, The University of North Carolina at Chapel Hill, Chapel Hill, NC 27599, USA; 5 Department of Biochemistry and Biophysics, The University of North Carolina at Chapel Hill, Chapel Hill, NC 27599, USA; 6 Department of Pharmacology, School of Medicine, The University of North Carolina at Chapel Hill, Chapel Hill, NC 27599, USA

**Keywords:** neurosteroid, inflammation, pregnenolone, allopregnanolone, cytokines

## Abstract

Pregnane neuroactive steroids, notably allopregnanolone and pregnenolone, exhibit efficacy in mitigating inflammatory signals triggered by toll-like receptor (TLR) activation, thus attenuating the production of inflammatory factors. Clinical studies highlight their therapeutic potential, particularly in conditions like postpartum depression (PPD), where the FDA-approved compound brexanolone, an intravenous formulation of allopregnanolone, effectively suppresses TLR-mediated inflammatory pathways, predicting symptom improvement. Additionally, pregnane neurosteroids exhibit trophic and anti-inflammatory properties, stimulating the production of vital trophic proteins and anti-inflammatory factors. Androstane neuroactive steroids, including estrogens and androgens, along with dehydroepiandrosterone (DHEA), display diverse effects on TLR expression and activation. Notably, androstenediol (ADIOL), an androstane neurosteroid, emerges as a potent anti-inflammatory agent, promising for therapeutic interventions. The dysregulation of immune responses via TLR signaling alongside reduced levels of endogenous neurosteroids significantly contributes to symptom severity across various neuropsychiatric disorders. Neuroactive steroids, such as allopregnanolone, demonstrate efficacy in alleviating symptoms of various neuropsychiatric disorders and modulating neuroimmune responses, offering potential intervention avenues. This review emphasizes the significant therapeutic potential of neuroactive steroids in modulating TLR signaling pathways, particularly in addressing inflammatory processes associated with neuropsychiatric disorders. It advances our understanding of the complex interplay between neuroactive steroids and immune responses, paving the way for personalized treatment strategies tailored to individual needs and providing insights for future research aimed at unraveling the intricacies of neuropsychiatric disorders.

## 1. Introduction

In recent years, the critical role of inflammation in neurological and psychiatric disorders has gained increasing recognition, stimulating interest in therapeutic agents capable of modulating these processes [1,2,3,4,5,6]. Among these agents, endogenous neuroactive steroids have emerged as significant candidates. Initially identified for their influence on gamma-aminobutyric acid type A (GABA_A_) receptors, such as allopregnanolone ((3α,5α)3-hydroxypregnan-20-one or 3α,5α-tetrahydroprogesterone (3α,5α-THP)), pregnanolone, and tetrahydrodeoxycorticosterone ([3α,5α]-3,21-dihydroxypregnan-20-one; 3α,5α-THDOC; THDOC), ongoing research has revealed their multifaceted functions and potential to influence central nervous system (CNS) disorders [7,8,9,10,11,12,13]. 

Neuroactive steroids are synthesized within both endocrine glands and the brain. In the brain, neurons are the primary producers of neurosteroids [14,15,16,17,18,19,20]. Neuroactive steroids, synthesized from cholesterol, can be classified into three categories: pregnane, androstane, and sulfated neuroactive steroids. Pregnanes, including allopregnanolone, pregnanolone, and 3α,5α-THDOC, act as positive modulators of GABA_A_ receptor subtypes. These compounds enhance inhibitory neurotransmission mediated by GABA_A_ receptors, leading to anxiolysis, sedation, anti-convulsant activity, and the enhancement of inhibitory circuits in the brain [21,22,23,24,25,26,27,28,29,30,31,32,33,34,35]. Importantly, their anti-inflammatory actions are distinct from their GABAergic mechanisms [36,37,38].

Preclinical and clinical studies have highlighted reduced levels of pregnane neuroactive steroids, such as pregnenolone and allopregnanolone, in conditions like stress, depression, post-traumatic stress disorder (PTSD), and alcohol use disorder (AUD) [39,40,41,42,43,44,45,46,47]. To address these deficits, researchers explored the therapeutic potential of pregnane steroids. Allopregnanolone and its precursors, pregnenolone and progesterone, have shown promise in animal models of AUD, chronic stress-induced depression, traumatic brain injury (TBI), multiple sclerosis (MS), and Alzheimer’s disease (AD) [48,49,50,51,52,53,54,55,56]. In clinical studies, progesterone has demonstrated efficacy in TBI and cocaine craving, while pregnenolone has shown benefits in alcohol and cannabis use disorders, and allopregnanolone has been effective in treating postpartum depression (PPD) [57,58,59,60,61,62,63,64,65,66].

Recent studies have highlighted the ability of pregnane neuroactive steroids to counteract inflammatory signals triggered by toll-like receptor (TLR) activation, reducing the production of inflammatory mediators. Clinical observations further support the therapeutic potential of compounds like brexanolone, a Food and Drug Administration (FDA)-approved intravenous formulation of allopregnanolone, in conditions such as PPD, attributed to their inhibition of TLR inflammatory pathways [36,37,54,64,67,68,69,70,71,72,73,74,75,76]. Furthermore, recent research has elucidated the trophic and anti-inflammatory properties demonstrated by pregnane neurosteroids, which stimulate the production of crucial trophic proteins and anti-inflammatory cytokines [77,78,79,80,81,82,83].

Moreover, research has examined the roles of androstane neuroactive steroids, encompassing dehydroepiandrosterone (DHEA) and androstenediol (ADIOL), as well as estrogens and androgens, in immune and neuroimmune regulation. The interplay between androstane neuroactive steroids and neuropsychiatric conditions is complex and influenced by factors such as sex, age, and individual differences [84,85,86,87,88,89,90,91,92,93,94,95,96,97,98,99,100,101,102,103,104,105]. Their impact on TLR signaling varies across specific contexts, diseases, and cell types [72,73,106,107,108,109,110,111,112,113,114,115,116,117,118,119,120,121,122,123,124,125,126,127,128,129,130,131,132,133,134,135,136,137,138]. 

TLRs, essential for pattern recognition, detect diverse molecular signatures, whether located on cell membranes or within endosomes, initiating signaling pathways that culminate in the expression of cytokines, chemokines, and interferons (IFN) [139,140,141,142]. Within the CNS, microglia, neurons, astrocytes, and oligodendrocytes express various TLR subtypes, orchestrating intricate neuroimmune signaling [143,144,145,146,147]. Significantly, even in cases of mild neuropathological conditions, communication among neurons and glia is characterized by the upregulation of TLR ligands. This upregulation leads to the activation of inflammatory TLR signaling pathways, subsequently resulting in the overexpression of pro-inflammatory mediators [146,148]. Excessive TLR signaling has been implicated in various neuroinflammatory conditions, including depression, substance use disorders, TBI, neurodegenerative diseases, and epilepsy [64,74,76,146,147,148,149,150,151,152,153,154,155,156,157,158,159,160,161,162,163,164,165,166,167,168,169,170,171,172,173,174,175,176,177,178,179,180,181,182,183,184,185,186,187]. These findings suggest potential therapeutic avenues for neurosteroids in mitigating neuroinflammation and neurodegenerative processes caused by TLR overactivation.

This review focuses on the role of endogenous neuroactive steroids in inflammation in both peripheral and brain contexts within neurological and psychiatric disorders. We delve into how these steroids, particularly pregnane neuroactive steroids, possess anti-inflammatory properties that operate independently of their effects on GABA_A_ receptors. This suggests potential mitigation of excessive TLR signaling and the related inflammatory and neuroinflammatory conditions. Additionally, we review the involvement of androstane neuroactive steroids in the regulation of inflammation and neuroinflammatory processes. The complex interplay between these neuroactive steroids and immune responses underscores their therapeutic potential in addressing a broad spectrum of neurological conditions, particularly through modulation of TLR signaling pathways.

## 2. Neurosteroids and Neuroimmune Regulation

### 2.1. Neurosteroids: An Overview and Classification

Neuroactive steroids are a class of endogenous steroids synthesized de novo within the CNS, independently of the endocrine gland steroidogenesis [23,49]. In fact, the presence of the enzymes necessary for the in-situ synthesis of neurosteroids has been found in several brain regions [9,17,22,23,188,189,190,191]. In the brain, neurons are the primary producers of neurosteroids such as pregnenolone, progesterone, and allopregnanolone [14,15,16,17,18,19]. While the capacity for de novo neurosteroidogenesis has been demonstrated in primary glial cultures from rat brains and human microglial cell lines [20,192], research suggests that in the adult mouse brain, glial cells lack the capacity to produce neurosteroids. Studies have shown that the enzymes 5α-reductase and 3α-hydroxysteroid dehydrogenase (3α-HSD), essential for the sequential synthesis of allopregnanolone and 3α,5α-THDOC from progesterone or deoxycorticosterone, respectively, are absent in glial cells. Specifically, there is no evidence of their presence in S100 calcium-binding protein B (S100β)- or glial fibrillary acidic protein (GFAP)-positive cells, indicating their absence in glial populations across various brain regions. Instead, these enzymes are predominantly localized in principal output neurons, including both glutamatergic and GABAergic neurons, distributed throughout diverse brain regions such as the cortex, hippocampus, olfactory bulb, thalamus, amygdala, striatum, and cerebellum [17]. These findings underscore the significance of both locally synthesized neurosteroids and those entering the brain from circulation in regulating brain function.

Neurosteroidogenesis begins with cholesterol or other steroidal precursors. The precursors are synthesized in the brain and systemically derived from steroids that cross the blood–brain barrier. A crucial step in steroidogenesis is the transportation of cholesterol within the mitochondria [193]. Here, cholesterol is converted to pregnenolone, the precursor of all steroids. Pregnenolone is then converted to progesterone, then other neurosteroids via progressive A-ring reductions [9,188]. Steroidogenesis involves several enzymes, protein transporters, redox partners, and cofactors. These mostly include forms of cytochrome P450 or hydroxysteroid dehydrogenases.

**Figure 1:**
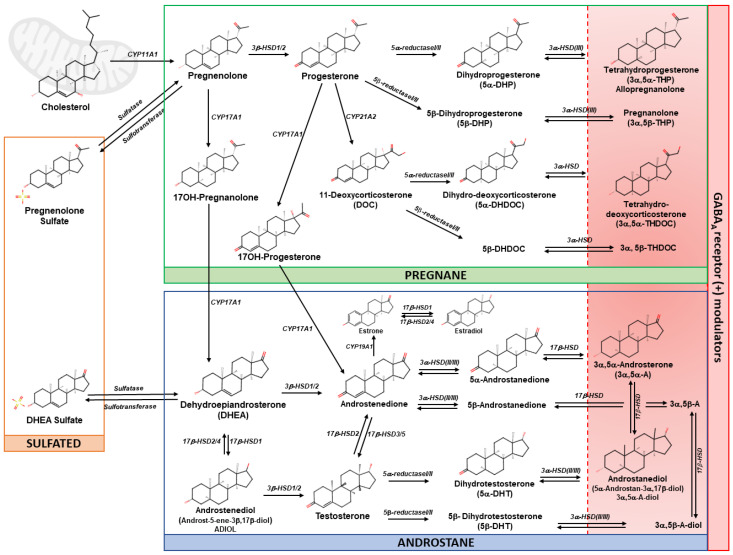
Neurosteroid biosynthesis and classification. Neurosteroid synthesis begins with the translocation of cholesterol into the mitochondria, where it is metabolized into pregnenolone by the cytochrome P450scc, a mitochondrial cholesterol side-chain cleavage enzyme (CYP11A1). Pregnenolone undergoes further conversions. It is transformed into progesterone by 3β-HSD 1/2 (3β-hydroxysteroid dehydrogenase). Progesterone can then be converted into 5α- or 5β-dihydroprogesterone (5α-DHP/5β-DHP) by 5α/β-reductase type I/II. 5α/β-DHP can be reduced to allopregnanolone (3α,5α-THP) or pregnanolone (3α,5β-THP) by the 3α-hydroxysteroid dehydrogenase III (3α-HSD) enzyme. Allopregnanolone can be reconverted into 5α-DHP or 5β-DHP. Progesterone can also be metabolized into 11-deoxycorticosterone (DOC) by the cytochrome P450 21-hydroxylase (CYP21A2), and further converted into 5α-dihydrodeoxycorticosterone (5α-DHDOC) or 5β-dihydrodeoxycorticosterone (5β-DHDOC) by the 5α- or 5β-reductases, respectively. 5α-DHDOC can be reduced into 3α,5α-THDOC (3α,5α-tetrahydro-deoxycorticosterone) and reconverted into 5α-DHDOC by the 3α-HSD enzyme. Likewise, 5β-DHDOC can be reduced into 3α,5β-THDOC (3α,5β-tetrahydrodeoxycorticosterone) and reconverted into 5β-DHDOC by the 3α-HSD enzyme. To form androstane steroids, pregnenolone can also be converted by the cytochrome P450-17A1 (CYP17A1) into 17OH-pregnanolone, then into dehydroepiandrosterone (DHEA). DHEA can be metabolized by 3β-HSD 1/2 into androstenedione, and then into estrone by the enzyme aromatase (CYP19A1). Estrone can be further converted into estradiol by 17β-HSD 1 (17β-hydroxysteroid dehydrogenase). Estradiol can be reconverted into estrone by 17β-HSD 2/4. The same enzyme, 17β-HSD 2/4, also converts DHEA into androstenediol (5-androstenediol, also known as androst-5-ene-3β,17β-diol; ADIOL). Androstenediol can be reconverted into DHEA by 17β-HSD 1 and further converted into testosterone by 3β-HSD 1/2. Moreover, androstenedione can be metabolized into testosterone by 17β-HSD 2 and reconverted into androstenedione by 17β-HSD 3/5. Alternatively, androstenedione can be converted to androstanedione by the 5α- or 5β-reductase enzymes and 3α-HSD to form 5α- or 5β-androsterone. Testosterone can be converted into estradiol by the aromatase (CYP19A1) or into dihydrotestosterone (5α-DHT or 5β-DHT) by the 5α- or 5β-reductase enzymes. Finally, 5α-DHT can be converted into androstanediol (3α-androstanediol also known as 5α-androstane-3α,17β-diol; 3α-diol) and reconverted into 5α-DHT by the 3α-HSD II/III enzymes. Pregnenolone and DHEA can also be converted into pregnenolone sulfate (PS) and DHEA sulfate (DHEAS) by the sulfatase enzyme and reconverted into pregnenolone and DHEA by removing the sulfate group with the sulfotransferase enzyme. Allopregnanolone, pregnanolone, 3α,5α- or 3α,5β-THDOC, 3α,5α- or 3α,5β-androsterone and 3α,5α- or 3α,5β-androstanediol all act as positive modulators of GABA_A_ receptors; however, the pregnane derivatives have much higher affinity and potency than the androstane derivatives.

Based on their molecular structure, neuroactive steroids can be classified as follows:

1. Pregnane steroids, derived from progesterone, such as allopregnanolone, pregnanolone and 3α,5α-THDOC;
2. Androstane steroids, derived from DHEA and testosterone, such as ADIOL and androstanediol;
3. Sulfated steroids, such as pregnenolone sulfate (PS) and dehydroepiandrosterone sulfate (DHEAS) (Figure 1).

Pregnane steroids are derived from progesterone, which originates from pregnenolone after its conversion from cholesterol by a dehydrogenase (Figure 1). Progesterone undergoes sequential A-ring reductions to yield allopregnanolone. This specific modification of the steroid structure significantly alters its biological activity by reducing the double bond in the A-ring, resulting in the formation of various pregnane derivatives. Additionally, another metabolite of progesterone, deoxycorticosterone, is reduced to form 3α,5α-THDOC. The biosynthesis of androstane steroids, primarily derived from DHEA and testosterone, involves similar A-ring reduction processes, producing products such as ADIOL, estradiol, and androstanediol (3α-diol) [194]. ADIOL, acting as an intermediate in the biosynthesis of testosterone from DHEA, requires the enzymatic activities of 17β-hydroxysteroid dehydrogenases (17β-HSD) [195]. It features a saturated A ring, an unsaturated bond in the B ring between C5 and C6, and a C19-methyl group [196]. Sulfated steroids are derived from the sulfation of specific precursors. For instance, pregnenolone sulfate is produced from the sulfation of pregnenolone [197]. Similarly, DHEA, a metabolite of 17OH-pregnanolone, is converted into DHEAS through sulfation [198,199].

The terminology for the neuroactive steroids was established in the late 1980s, during a period when the field was just beginning to unravel the direct influence of allopregnanolone on GABA_A_ receptors [7,8,9]. Similarly, investigations unveiled the direct impact of pregnenolone on glutamate receptors [10]. Ongoing studies on neurosteroids such as allopregnanolone and pregnenolone have revealed a diverse array of action sites, suggesting that their multifaceted functions collectively contribute significantly to their neurological effects and potential to influence CNS disorders [11,12,13]. 

Many neurosteroids can exert their effects directly or through their metabolites, influencing various genomic and non-genomic mechanisms within the brain. For example, pregnenolone undergoes metabolic conversion to conventional steroids, such as progesterone, estrogen, androgen, glucocorticoid and mineralocorticoid, which are then mediated by their respective nuclear receptors [200,201]. Beyond its well-established role as a neurosteroid precursor, pregnenolone also has the capacity to directly interact with molecular targets. Notably, it can bind to microtubule-associated protein 2 (MAP2) and the cannabinoid receptor type 1 (CB1) [202,203]. In addition, estradiol and allopregnanolone can bind to and activate xenobiotic receptors, including the pregnane X receptor [204,205]. In the cytosol, estradiol binds to nuclear steroid receptors that are anchored to chaperone proteins. This binding releases the receptors from their anchoring, facilitating their dimerization [206]. Subsequently, the receptor complex migrates into the nucleus where, in conjunction with transcriptional coactivators, it binds to specific DNA sequences, thereby activating gene transcription [206]. This activation of genomic pathways by neurosteroids leads to trophic effects on neurons and glia, as well as modulation of neurotransmission and the synthesis of proteins that enhance neuroplasticity [207,208]. 

In contrast to the slow effects of steroid hormones via intracellular steroid receptors, neurosteroids can also rapidly modulate brain excitability through non-genomic actions on neurotransmitter receptors. GABA_A_ receptors are the primary mediators of inhibitory transmission in the brain. These receptors are heteropentamers, typically composed of α, β, and either γ or δ subunits, forming chloride ion channels [209]. GABA_A_ receptors facilitate two types of inhibitory neurotransmission: synaptic (phasic) and extrasynaptic (tonic) inhibition. Neurosteroids with GABAergic activity modulate both types, influencing phasic and tonic inhibition. These neurosteroids bind to specific sites on the GABA_A_ receptors, located at the interface of the α and β subunits of synaptic receptors [210], distinct from the binding sites of GABA, benzodiazepines and barbiturates. Allopregnanolone and 3α,5α-THDOC are notably potent in most GABA_A_ receptor subtypes [211], with enhanced potency at extrasynaptic receptors containing the δ subunit [32,212,213]. Exposure to neurosteroids prolongs the mean open time of the GABA_A_ receptor chloride channels, increasing chloride current, leading to membrane hyperpolarization and a subsequent reduction in neuronal excitability. At high concentrations, neurosteroids like allopregnanolone can directly activate GABA_A_ receptors even in the absence of GABA [214]. Neurosteroid modulation depends on specific structural features, including a 3α-hydroxy group on the A-ring and a hydrogen bond-accepting group on the D ring, found at either C20 or C17 of the steroid side chains [9,25,215]. Sulfated neurosteroids like PS and DHEAS act as weak non-competitive antagonists of GABA_A_ receptors and potent allosteric modulators of N-methyl-D-aspartate (NMDA) receptors [216,217,218,219,220]. PS can also directly affect α-amino-3-hydroxy-5-methyl-4-isoxazolepropionic acid (AMPA) receptors [221]. PS and DHEAS are sigma receptor agonists, whereas progesterone acts as a potent antagonist [222]. 

In summary, 3α-hydroxy-pregnane derivatives like allopregnanolone, pregnanolone, and 3α,5α-THDOC, along with various synthetic compounds, act as positive modulators of GABA_A_ receptor subtypes [24,25,26,27,28,29,30]. These compounds enhance inhibitory neurotransmission mediated by GABA_A_ receptors, leading to anxiolysis, sedation, anti-convulsant activity, anti-depressant activity, and the enhancement of inhibitory circuits in the brain [31,32,33,34,35,223]. Conversely, neurosteroids like PS and DHEAS are considered excitatory, known to enhance memory and produce anxiogenic effects [224]. The ability of neurosteroids to modulate excitatory and inhibitory circuits in the brain is essential for maintaining homeostasis and plays a role in neuropsychiatric diseases where this balance between excitation and inhibition is disrupted.

For many years, neurosteroids have been proposed as a therapy to restore altered GABAergic transmission in conditions such as depression, PTSD, PPD, anxiety, and substance use disorders [11,12,13,61,225,226]. These neuropsychiatric conditions share, among other features, a dysregulation in hypothalamic–pituitary–adrenal (HPA) axis activity [44,46,227,228,229,230,231,232,233], likely due to aberrant GABAergic signaling [234,235]. For example, patients with PTSD typically exhibit elevated levels of corticotropin-releasing factor (CRF) and a reduction in allopregnanolone levels in their cerebrospinal fluid (CSF) [236]. Animal models of affective disorders, such as social isolation in rats and mice, often display altered levels of HPA axis biomarkers and reduced levels of brain and plasma allopregnanolone [237,238,239,240,241]. Likewise, patients with AUD also exhibit elevated levels of CRF and cortisol along with blunted stress responses and reductions in serum allopregnanolone [47,228,242]. Similar results are found in animal models of ethanol dependence where CRF is elevated, various neurosteroids are depleted, and stress responses are blunted (see Morrow et al., 2020, for review [12]). 

Although major depressive disorder (MDD) presents with various symptoms and affects multiple systems, dysregulation of HPA axis activity is a common feature [243]. Indeed, individuals with MDD frequently demonstrate low levels of allopregnanolone in both plasma and CSF [244,245,246]. Women experiencing PPD also show alterations in GABA and allopregnanolone levels [247,248] as well as HPA axis hypoactivity and hormonal imbalances [249,250]. Animal studies further support these findings, revealing that mice lacking the δ subunit in GABA_A_ receptors exhibit behaviors akin to PPD, disinhibited CRF signaling, and elevated corticosterone levels during the postpartum period [251,252,253]. 

Over the past 30 years, evidence has emerged indicating that allopregnanolone and other neurosteroids can normalize dysfunction within the HPA axis. Administration of allopregnanolone has been shown to reduce stress-induced increases in CRF expression [254], while both allopregnanolone and 3α,5α-THDOC administration have prevented stress-induced elevations in adrenocorticotropic hormone and corticosterone levels when administered before stress induction in rats [255,256]. These findings suggest that treatment with allopregnanolone and other neurosteroids may hold therapeutic promise for neuropsychiatric disorders characterized by dysregulation in HPA axis and GABAergic signaling. 

Most relevant to this review, neuropsychiatric diseases have been increasingly associated with neuroinflammation [257,258]. Recent research has uncovered the anti-inflammatory effects of neurosteroids in both the brain and peripheral tissues. Notably, these effects influence TLR activation and signaling, resulting in the inhibition of pro-inflammatory mediators and the enhancement of anti-inflammatory mediators. Importantly, these effects occur independently of neurosteroid actions on GABA_A_ receptors [36,37,64,67,68,80].

### 2.2. Toll-like Receptor Signaling and Neuroimmune Regulation

TLRs, crucial for pattern recognition, detect various molecular signatures such as pathogen-associated molecular patterns, danger-associated molecular patterns, and microbiome/microbe-associated molecular patterns. These receptors are situated either on the cell membrane (e.g., TLR1, TLR2, TLR4, TLR5, TLR6, TLR11, TLR12) or within endosomes (e.g., TLR3, TLR7, TLR8, TLR9, TLR13). Once these molecular patterns are recognized, TLRs initiate signaling pathways that lead to the expression of cytokines, chemokines, and IFNs [139,140,141,142]. Typically, TLRs (excluding TLR3 and TLR4) signal primarily through myeloid differentiation primary response 88 (MyD88)-dependent pathways. However, TLR3 exclusively signals through toll/interleukin-1 receptor domain-containing adapter-inducing IFN-β (TRIF)-dependent pathways, while TLR4 can be activated through both MyD88- and TRIF-dependent pathways. The MyD88-dependent TLR4 pathway functions on the cell membrane and entails the recruitment of co-receptors: cluster of differentiation 14 protein (CD14), facilitating the recognition of lipopolysaccharide (LPS), and myeloid differentiation protein 2 (MD2), aiding in the recognition and binding of LPS. Additionally, adaptor molecules such as MyD88 and toll/interleukin-1 receptor domain-containing adapter protein (TIRAP) are involved in this pathway [140,141]. Conversely, the TRIF-dependent TLR4 pathway occurs within endosomes and is initiated by adaptors TRIF and TLR4-specific TRIF-related adapter molecule (TRAM) [259,260,261]. TIRAP facilitates the transmission of signals from TLR4 to MyD88, while TRAM facilitates the transmission of signals from TLR4 to TRIF. Upon engagement of TLRs, the myddosome complex is formed, consisting of MyD88, interleukin-1 receptor-associated kinase (IRAK) 1, and IRAK4. Activation of IRAK1 triggers the activation of tumor necrosis factor (TNF) receptor-associated factor 6 (TRAF6) through lysine 63/K63 linked polyubiquitination on both TRAF6 itself and transforming growth factor beta-activated kinase 1 (TAK1). Activation of TAK1 subsequently leads to the activation of the inhibitor of kappa-B (IκB) kinase (IKK) complex, resulting in the phosphorylation and subsequent degradation of IκB proteins. This allows for the nuclear translocation of nuclear factor kappa-B (NF-κB) transcription factors and the initiation of gene transcription. Furthermore, activation of TAK1 leads to the activation of mitogen-activated protein kinases (MAPKs) (e.g., extracellular signal-regulated kinase (ERK), c-Jun N-terminal kinase (JNK), p38), which in turn activate or phosphorylate various transcription factors, promoting the production of pro-inflammatory mediators [139,140,141,142,262]. 

In the CNS, various types of TLRs are present. Microglial cells express TLR1 through TLR9, neurons express TLR3, TLR4, TLR7, TLR8, and TLR9, astrocytes express TLR2, TLR3, and TLR9, while oligodendrocytes express TLR2 and TLR3 [143,144,145,146,147]. Interactions among microglia, neurons, astrocytes, and oligodendrocytes are pivotal in neuroimmune signaling, as highlighted by previous studies [146,263,264]. Microglia, the resident immune cells, respond to neuronal signals by engaging in immune responses, phagocytosis, and cytokine/chemokine secretion. Neurons contribute to maintaining microglial quiescence through regulatory signals [265,266]. Imbalances due to disease or injury trigger microglial activation, influencing tissue repair or neurotoxicity, which is modulated by various stimuli and the microenvironment [267]. Astrocytes release molecules affecting synaptic function and immune responses [268,269], while oligodendrocytes interact with microglia via immune receptor expression [270]. Additionally, cross-communication among brain cells involves the up-regulation of TLR ligands, activation of TLR signaling pathways, and subsequent over-expression of chemokines and cytokines, such as monocyte chemoattractant protein-1 (MCP-1), TNF-α, interleukin (IL)-1β, IL-6, high mobility group box 1 (HMGB1), and their receptors, which regulate distinct neuroimmune responses and cell-to-cell interactions [146,148].

Excessive inflammatory TLR activation is implicated in the development of various neuropsychiatric disorders. Activation of TLR pathways in glial cells, infiltrating lymphocytes, and neurons in the brain leads to the production of pro-inflammatory molecules, contributing to neuronal damage [169,173]. Stimulating the innate immune response with TLR4 agonist LPS in rodents results in neuronal injury, oligodendrocyte loss, hypomyelination, and periventricular cysts, reminiscent of periventricular leukomalacia and MS. Conversely, animals lacking functional TLR4 showed resistance to neurodegeneration [171,172]. Moreover, inhibition of the HMGB1/TLR4/MyD88 signaling pathway reduced levels of inflammatory cytokines and elevated brain-derived neurotrophic factor (BDNF) levels in the rat brain [174]. Additionally, neurodegeneration occurs due to the activation of the neuronal TLR7 pathway by the miRNA let-7 [170]. 

The activation of inflammatory responses through TLR signaling closely correlates with brain tissue damage and neurological impairment following TBI [167]. Inhibiting TLR4 resulted in enhanced neurological outcomes following TBI in mice, potentially attributable to heightened levels of anti-inflammatory monocytes and diminished IFN regulatory factor (IRF) 7 during acute inflammation, followed by a reduction in apoptosis and M2 microglial expression during chronic inflammation [168]. Moreover, the attenuation of TLR4/NF-kB inflammatory signaling after TBI in rats is associated with a notable reduction in neuronal cell apoptosis [166]. 

TLRs play a crucial role in modulating ischemic brain damage post-stroke [177,178]. In neonatal hypoxic-ischemic brain damage in rats, inhibition of the TLR4/MyD88 signaling pathway activation led to improved hippocampal structure and decreased inflammatory markers, indicating neuroprotective effects [179]. Additionally, TLR4-deficient mice exhibited reduced infarctions and a dampened inflammatory response following ischemic insult, underscoring the significance of TLR4 signaling in ischemic brain damage and inflammation [175]. 

TLR signaling emerges as a pivotal factor in epilepsy pathogenesis, evident from brain inflammation observed in both clinical cases and experimental models [185,186,187,271,272]. Of particular significance is the interaction between HMGB1 and TLR4 in this pathway, which plays a pivotal role in seizure generation and perpetuation. This signaling cascade triggers rapid alterations in NMDA receptors and induces long-term changes in seizure thresholds. Encouragingly, antagonists targeting the HMGB1/TLR4 pathway have shown promise in mitigating epilepsy-related pathology [186,271,273]. Furthermore, TLR3 deficiency has been shown to reduce spontaneous recurrent seizures, microglial activation, and levels of proinflammatory cytokines TNF-α and IFN-β following status epilepticus. Additionally, TLR3-deficient mice demonstrated enhanced survival rates post-status epilepticus [274]. 

The overactivation of TLRs significantly impacts the interplay of the innate immune system with neurotransmission, neuroendocrine functions, and stress-induced adaptations, thereby contributing to MDD and stress [152,153]. Among TLRs, TLR4 stands out for its prominent involvement in stress-induced neuroinflammatory responses and subsequent behavioral alterations. Moreover, the activation of the TLR4/MyD88/NF-κB pathway has been linked to chronic unpredictable mild stress in rat models [154,156]. In a mouse model of hippocampal neuroinflammation, the activation of the TLR4/TRAF6/NF-κB signaling pathway, triggered by LPS, emerges as a significant contributor to depressive-like behaviors [155]. Notably, recent research has unveiled a connection between LPS-induced TLR4 activation and depression in mice, particularly concerning deltamethrin-induced disruption of the gut–brain axis [151]. Additionally, inflammation resulting from the activation of both the TLR4 and TLR7 pathways has been observed in individuals experiencing PPD [64]. 

In the context of substance use disorders, TLRs are triggered and upregulated within the CNS in response to endogenous innate immune agonists like HMGB1 and miRNAs, due to exposure to substances such as alcohol and other addictive drugs. Specifically, in individuals with AUD, TLRs, particularly TLR2-9, and HMGB1 are induced, subsequently activating NF-κB. This activation leads to the upregulation of proinflammatory cytokines, chemokines, and their receptors. Ultimately, these molecular changes contribute to epigenetic modifications, neurodegeneration, and disruptions in synaptic plasticity, which play pivotal roles in the manifestation of cognitive and affective impairments observed in individuals with alcohol addiction [157,158,159,160,162,164,275]. 

Binge drinking, often intertwined with cognitive impulsivity, anxiety, and smoking habits, triggers neuroimmune signaling through TLR4. In the brain, this pathway’s activation is facilitated by the GABA_A_ α2 subunit protein, when it operates independently, separate from the GABA_A_ receptor. Consequently, it initiates cyclic adenosine monophosphate response element-binding protein (CREB) activation and the upregulation of CRF, tyrosine hydroxylase (TH), and MCP-1. Encouragingly, gene therapy aimed at modulating neuronal TLR4, α2, or MCP-1 has shown promise in mitigating binge drinking, nicotine sensitization, and cognitive impulsivity, highlighting their potential as key regulators of these behaviors [147,148,161,163,165].

Interestingly, TLRs are implicated in the immunopathogenesis of schizophrenia. Drug-naïve patients with schizophrenia exhibited increased TLR4 mRNA levels and unaltered TLR3 mRNA levels in peripheral blood mononuclear cells (PBMC), alongside elevated TLR4 and TLR8 mRNA levels and reduced TLR3 mRNA levels in white blood cells compared to healthy controls [180,181]. Additionally, the correlation between TLRs and complement factors suggests a coordinated immune response in schizophrenia. Higher TLR8 mRNA levels were inversely associated with cortical thickness of the cingulate gyrus, hinting at a potential link between TLR activation and structural brain changes in schizophrenia [181]. Furthermore, no significant changes in TLR3 and TLR4 gene expression in PBMCs were observed after three months of antipsychotic medication [180]. 

TLRs emerge as pivotal contributors to neuropathic pain [182,183]. Studies revealing a causal relationship demonstrate a significant association between TLR4 and neuropathic pain. Mouse models lacking functional TLR4 following L5 nerve transection exhibit reduced behavioral hypersensitivity, alongside diminished expression of spinal microglial markers and proinflammatory cytokines. Similarly, rats with downregulated TLR4 post L5 nerve transection display comparable results [276]. Furthermore, CD14’s significant involvement in the LPS-TLR4 signaling pathway in nerve injury-induced neuropathic pain is underscored, as evidenced by decreased behavioral sensitivity in CD14 knockout mice following L5 spinal nerve transection [277]. Moreover, heightened TLR3 expression emerges as a crucial factor in neuropathic pain onset in rats subjected to the L5 spinal nerve ligation model. This increased TLR3 activity influences autophagy pathways within affected neurons, contributing to neuropathic pain development [278]. Similarly, augmented TLR7 expression in dorsal root ganglion neurons exacerbates neuropathic pain, while inhibiting this upregulation alleviates pain hypersensitivity and reduces inflammatory markers in the dorsal horn. Conversely, TLR7 overexpression induces pain sensitivity [279]. Additionally, activation of TLR8 in dorsal root ganglion neurons fosters ERK activation, prompting the production of inflammatory mediators and heightened neuronal hyperexcitability, thereby contributing to neuropathic pain post spinal nerve ligation [280]. Notably, blocking the TLR2/MyD88/NF-κB pathway in microglia within the spinal cord dorsal horn confers neuroprotection, offering sustained relief from neuropathic pain through the secretion of tumor necrosis factor-stimulated gene 6 protein [281]. 

Although TLRs are commonly linked to inflammation, evidence suggests they can exhibit both pro-inflammatory and anti-inflammatory functions, influenced by factors such as cell type, sex, subcellular compartmentalization of TLR signaling, ligand type, and involvement of co-receptors [80,282,283,284]. For instance, TLR2 demonstrates anti-inflammatory properties when exposed to polysaccharide A from Bacteroides fragilis, triggering IL-10 and IFN-γ secretion, which are protective against viral encephalitis [285,286,287]. Additionally, TLR2 plays a vital role in the anti-inflammatory response to Listeria monocytogenes infection, with endosomal TLRs predominantly responsible for immune suppression against phagosome-confined bacteria [288]. In glucocorticoid treatment and the resolution phase of inflammation, TLR2 upregulation occurs, facilitated by glucocorticoid receptor-dependent mechanisms. This upregulation leads to the production of soluble TLR2, known to antagonize TLR2-dependent actions, and its presence in extracellular vesicles acts as decoy receptors, dampening inflammatory responses [289]. On the other hand, TLR4’s anti-inflammatory effects rely on its intracellular compartmentalization, orchestrated by the p110δ isoform of phosphoinositide 3-kinase (PI3K). This process guides TLR4 from an initial plasma membrane complex associated with TIRAP-MyD88 to a later endosomal complex involving TRAM-TRIF, triggering the production of anti-inflammatory type I IFNs and IL-10 [80,283,284]. Mechanistically, p110δ reduces the abundance of the TIRAP-anchoring lipid phosphatidylinositol-(4,5)-bisphosphate (PtdIns(4,5)P2) on the plasma membrane. This action prompts the endocytosis of CD14-TLR4 by mobilizing Ca^2+^. The subsequent turnover of PtdIns(4,5)P2 frees TIRAP into the cytoplasm, where it undergoes degradation by calpains and the proteasome. Disabling p110δ shifts the balance toward proinflammatory early signaling, heightening sensitivity to endotoxins [283,284]. Moreover, in male alcohol-preferring rat brains, neurosteroid allopregnanolone enhances the activation of the endosomal anti-inflammatory TLR4/TRIF pathway, while inhibiting it in females, suggesting a sex-dependent mechanism [80]. Additionally, TLR4 recognizes specific molecules from probiotics, like exopolysaccharide (EPS) from Bacillus subtilis, to address intestinal inflammatory diseases. EPS activates the anti-inflammatory TLR4 pathway, leading to the expression of the immunosuppressive enzyme indoleamine 2,3-dioxygenase in dendritic cells, inhibiting T cell proliferation via the kynurenine/aryl hydrocarbon receptor circuit. Notably, unlike LPS, EPS administration does not induce the release of inflammatory cytokines, emphasizing the distinct effects of different TLR4 agonists [290]. Lastly, TLR9 signaling regulates anti-inflammatory responses in lupus independently of MyD88, while TLR10 upregulation by vitamin D promotes the expression of anti-inflammatory cytokines in microglial cells, favoring M2 polarization [291,292,293].

In summary, TLRs play a crucial role in recognizing various molecular patterns, triggering signaling pathways that lead to the expression of cytokines, chemokines and IFNs. Overactive TLRs have been associated with various neurological disorders, encompassing neurodegeneration, AUD, TBI, epilepsy, and psychiatric diseases. This emphasizes the importance of therapeutic approaches targeting TLR signaling, with an emphasis on inhibiting inflammatory pathways while enhancing anti-inflammatory mechanisms. Such interventions offer potential for safeguarding neurological health and managing neuroinflammatory disorders.

### 2.3. Mechanisms of Action of Pregnane Neuroactive Steroids on Toll-like Receptors

Pregnane neuroactive steroids demonstrate anti-inflammatory effects in both the brain and peripheral tissues. These effects impact TLR activation and signaling pathways, leading to the inhibition of pro-inflammatory mediators and the enhancement of anti-inflammatory mediators [36,37,64,67,68,69,71,80,294]. 

**Figure 2:**
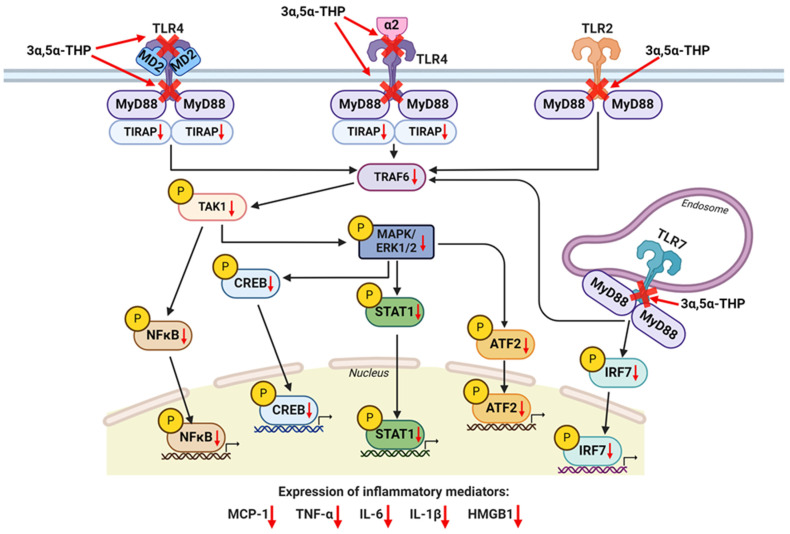
The schematic model illustrates the inhibitory effects of allopregnanolone (3α,5α-THP) on toll-like receptor (TLR) inflammatory signaling pathways. Black line arrows represent TLR inflammatory pathways, while red X-shapes or arrows indicate the impacts of 3α,5α-THP. 3α,5α-THP inhibits the binding of TLR4 with both myeloid differentiation protein 2 (MD2) and myeloid differentiation primary response 88 (MyD88), as well as the α2 subunit protein of the gamma-aminobutyric acid A receptors. The binding of TLR2 and TLR7 with MyD88 is also inhibited by 3α,5α-THP (indicated by red X-shapes), preventing TLR pathway activation/initiation. Additionally, 3α,5α-THP promotes the degradation of TLR4 adapter toll/interleukin-1 receptor domain-containing adapter protein (TIRAP) (indicated by red arrows). Consequently, these events lead to decreased levels of tumor necrosis factor receptor-associated factor 6 (TRAF6) and decreased activation of transforming growth factor beta-activated kinase 1 (TAK1), as evidenced by its decreased phosphorylation (indicated by a red arrow). The inhibition of TAK1 activation suppresses the activation of nuclear factor kappa-B (NF-κB) and mitogen-activated protein kinases (MAPK), such as extracellular signal-regulated kinases 1/2 (ERK1/2), thereby inhibiting the activation of various transcription factors including cAMP response element-binding protein (CREB), signal transducer and activator of transcription 1 (STAT1) and activating transcription factor 2 (ATF2). Inhibition of TLR7/MyD88 signaling also involves the inhibition of interferon regulatory factor 7 (IRF7) activation. These events collectively result in the decline in inflammatory mediators including monocyte chemoattractant protein-1 (MCP-1), tumor necrosis factor alpha (TNF-α), interleukin 6 (IL-6), interleukin 1 beta (IL-1β), and high mobility group box 1 protein (HMGB1). The schematic figure was created with BioRender.com.

Allopregnanolone reduces pro-inflammatory TLR signal activation through a highly specific mechanism (Figure 2). This mechanism involves the inhibition of crucial protein–protein interactions essential for TLR activation. Its role as an inhibitor is particularly pronounced in the activated TLR signaling pathways, while it leaves the steady-state, non-activated pathways unaffected. Allopregnanolone’s inhibitory action has been delineated in pro-inflammatory signaling triggered by TLR2, TLR4, and TLR7. This inhibition hinges on its ability to disrupt key protein binding steps, including those between TLR2 and MyD88, TLR4 and MD2, TLR4 and MyD88, and TLR7 and MyD88. These inhibitions have been observed both in the mouse macrophage RAW264.7 cell line and in rat brains [36,67].

Intriguingly, allopregnanolone also inhibits the binding of the GABA_A_ α2 subunit protein to TLR4 in the rat brain [36]. This is of significance as previous research has indicated that the α2 subunit protein binding to TLR4 is the essential binding step for TLR4 signal activation in the alcohol preferring (P) rat brain [147,161].

The disruption of these protein–protein interactions and allopregnanolone’s intervention leads to a substantial reduction in the activation of various downstream components within the TLR signaling pathways. These inhibitory effects encompass decreased levels of TRAF6, as well as reduced phosphorylation (p) or activation of TAK1 and ERK1/2. Additionally, transcription factors such as NF-κB, CREB, activating transcription factor (ATF)2, signal transducer and activator of transcription (STAT)1, and IRF7 exhibit diminished activity. Ultimately, this cascade of events results in decreased expression of pro-inflammatory factors, including MCP-1, HMGB1, TNF-α, IL-1β, and IL-6 (Figure 2) [36,37,67].

Importantly, allopregnanolone inhibition of TLRs specifically targets the MyD88-dependent pro-inflammatory pathways, leaving the TRIF-dependent TLR3 pathway unaffected [67]. Furthermore, these inhibitory effects are independent of GABA_A_ receptor activity and are replicated by pregnenolone, which lacks GABAergic activity. In addition, the GABAergic steroid 3α,5α-THDOC lacks many of the anti-inflammatory actions of allopregnanolone, although it does exhibit some influence on downstream TLR4 and TLR7 pathway members in cultured human monocyte-derived macrophages obtained from female, but not male donors [36,37]. 

In human macrophages, allopregnanolone consistently demonstrates its ability to inhibit TLR4 activation and the associated inflammatory pathway members CREB, STAT1, MCP-1 and TNF-α in cells from both male and female subjects. However, the inhibition of TLR7 activation appears to be sex-specific, primarily blocking STAT1, IRF7, IL-1β, and IL-6 elevations in macrophages from female donors. This finding suggests potential sex differences in the actions of pregnane neuroactive steroids on TLR7 receptors, warranting further investigation in future studies. 

Furthermore, other studies show that allopregnanolone inhibits the activation of TLR4 and TLR2 in both mouse macrophages and microglial cells by inducing the degradation of both TLR2 and TIRAP, leading to reduced TNF and IL-6 levels [68]. Notably, a similar impact of allopregnanolone on TIRAP degradation was observed in P rat brains [80].

**Figure 3:**
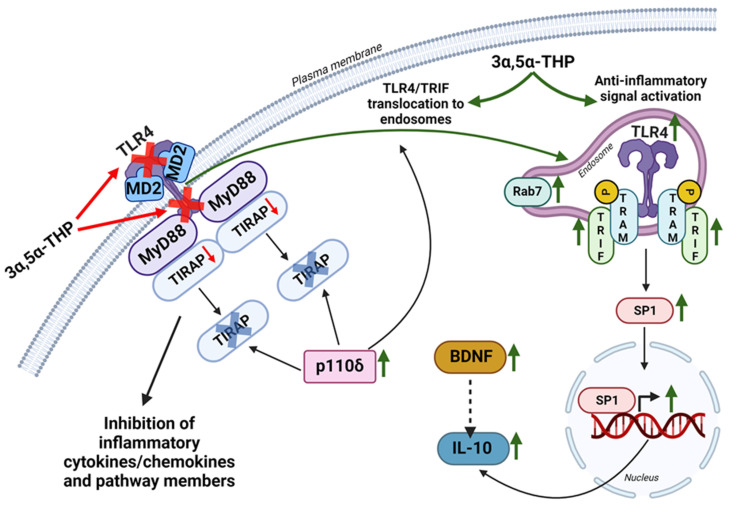
The schematic model illustrates how allopregnanolone (3α,5α-THP) induces endosomal toll/interleukin-1 receptor domain-containing adapter-inducing interferon-beta (TRIF)-dependent toll-like receptor 4 (TLR4) anti-inflammatory signaling, leading to elevated levels of interleukin-10 (IL-10). 3α,5α-THP facilitates the transition of TLR4 from the toll/interleukin-1 receptor (TIR) domain-containing adapter protein (TIRAP)-myeloid differentiation primary response 88 (MyD88)-associated plasma membrane complex to an endosomal TRIF-related adapter molecule (TRAM)-TRIF complex, initiating the activation of the endosomal anti-inflammatory TLR4-TRIF signal and subsequent IL-10 production. Mechanistically, 3α,5α-THP upregulates the p110δ isoform of phosphoinositide 3-kinase (PI3K), promoting the degradation of TIRAP and the release of TLR4 from the TIRAP-MyD88-associated plasma membrane complex, facilitating TLR4 translocation to endosomes. Additionally, 3α,5α-THP facilitates TRIF accumulation in endosomes. The release of TLR4 from the TIRAP-MyD88-associated plasma membrane complex may also result from direct 3α,5α-THP-induced inhibition of the binding between TLR4 and MyD88, and TLR4 and myeloid differentiation factor 2 (MD2). Furthermore, both the inhibition of binding and TIRAP degradation suppress inflammatory TLR4 pathway components, cytokines, and chemokines. 3α,5α-THP activates the anti-inflammatory endosomal TLR4-TRIF pathway by triggering an increase in phosphorylated TRAM, a specific marker for TLR4-TRIF pathway activation. The model also incorporates 3α,5α-THP-induced enhanced presence of transcription factor specificity protein 1 (SP1), leading to increased IL-10 production. Additionally, 3α,5α-THP upregulates brain-derived neurotrophic factor (BDNF) levels, potentially amplifying IL-10 production, and release. Furthermore, 3α,5α-THP stimulates the accumulation of endosomal Ras-related protein Rab7 (Rab7), which may significantly impact the equilibrium between pro-inflammatory and anti-inflammatory TLR4 signaling pathways. In the figure, an increase in protein levels is indicated by a green up-arrow, protein degradation by a blue X-shape, and inhibition of protein–protein binding by a red X-shape. The schematic figure was created with BioRender.com.

Additionally, allopregnanolone plays a crucial role in modulating the immune response by inducing anti-inflammatory signaling through the endosomal TRIF-dependent TLR4 pathway, but notably, this effect is observed in the male rat brain and not in the female rat brain (Figure 3) [80]. This process culminates in the elevation of IL-10, a central regulator of inflammation in both brain and peripheral tissues [295,296,297,298]. The underlying mechanism hinges on several crucial steps. Allopregnanolone triggers the translocation of TLR4 from the plasma membrane to endosomes, driven by the upregulation of the p110δ isoform of PI3K and the degradation of TIRAP. This results in the accumulation of TLR4 and TRIF, leading to the formation of a TLR4-TRIF complex within endosomes, subsequently activating the anti-inflammatory signaling cascade [80,283,284]. Allopregnanolone further enhances this pathway by increasing phosphorylated (activated) TRAM levels, resulting in the upregulation of transcription factor specificity protein 1 (SP1) and subsequent production of IL-10 [80]. Additionally, allopregnanolone upregulates BDNF levels, potentially amplifying IL-10 production, and release [77,79,80,299,300,301]. Furthermore, allopregnanolone stimulates the accumulation of endosomal Ras-related protein Rab7, which holds the potential to influence the equilibrium between pro-inflammatory and anti-inflammatory TLR4 signaling pathways within the male rat brain [80]. The mechanism underlying the sex-specific effects of allopregnanolone on IL-10 remains unclear and might potentially be influenced by varying sensitivities to allopregnanolone doses. 

Interestingly, allopregnanolone, along with 3α,5α-THDOC and pregnenolone, can enhance the expression of the anti-inflammatory chemokine fractalkine (CX3CL1) within the alcohol-preferring P rat brain [302]. This is noteworthy, especially considering that fractalkine is innately downregulated in these rats. The downregulation of fractalkine can be attributed to inflammatory TLR4 signaling, which results in the dysregulation of anti-inflammatory modulators like fractalkine in favor of inflammatory modulators like MCP-1. The data suggest that allopregnanolone may contribute to maintaining a homeostatic balance between inflammatory and anti-inflammatory modulators within the brain, potentially influencing susceptibility to inflammatory brain diseases, including neuropsychiatric conditions [146,302]. 

Like allopregnanolone, pregnenolone, which lacks innate GABAergic properties, plays a role in reducing pro-inflammatory TLR signal activation by inhibiting the binding of TLR4 with MD2 and TLR4 with MyD88. This indicates that pregnane neuroactive steroids’ inhibitory effects on pro-inflammatory TLR signaling are not dependent on GABAergic mechanisms [36]. Furthermore, while pregnenolone serves as a precursor for allopregnanolone in steroidogenic cells, the observed inhibitory effects of pregnenolone in the mouse macrophage RAW264.7 cell line were not attributed to its conversion to allopregnanolone. This conclusion is supported by the detection of minimal conversion (less than 0.1%) in these cells [36].

High-throughput mass spectrometry identified 11 specific pregnenolone-binding proteins in Th2 CD4+ immune cells and 25 in CD8+ immune cells, located in mitochondria and the endoplasmic reticulum, with a significant presence in membranes, highlighting pregnenolone’s non-genomic activity [303,304]. Pregnenolone possesses the capability to directly interact with MAP2 and CB1 receptors in the brain [202,203]. Furthermore, it appears to attenuate inflammatory TLR signaling by modulating CB1 receptors, potentially contributing to anti-inflammatory effects [305,306]. However, future research is needed to elucidate whether pregnenolone directly binds to components of TLR signaling pathways and TLRs themselves, and if it indeed suppresses inflammatory TLR signaling via CB1 receptors.

Furthermore, pregnenolone exhibits another facet of its anti-inflammatory action by promoting the ubiquitination and degradation of TLR2 and TIRAP [68]. In the RAW264.7 cells, pregnenolone selectively inhibits the formation of the TLR4/MD2 complex, a pivotal step in initiating the TLR4-mediated signaling pathway. This inhibition results in a significant reduction in the activation of various downstream components within the TLR4 signaling pathway when the cells are exposed to agonists such as LPS. These inhibitory effects involve a decrease in TRAF6 levels and a reduction in the activities of TAK1, NF-κB, and CREB. Consequently, there is a decreased expression of pro-inflammatory mediators, including MCP-1, HMGB1, and TNF-α. Importantly, this inhibition by pregnenolone is specific to the LPS-activated TLR4 pathways and does not affect these proteins in non-activated cells [36].

Furthermore, in experiments involving mouse and human macrophage cell lines, along with mouse microglial cells, pregnenolone demonstrates its ability to suppress the secretion of pro-inflammatory cytokines, particularly TNF-α and IL-6, in response to TLR4 and TLR2 signal activation. Pregnenolone achieves this by promoting the ubiquitination and subsequent degradation of TIRAP and TLR2. Additionally, pregnenolone triggers the activation of the microtubule plus end-binding protein, further enhancing the degradation of TIRAP and the suppression of TLR4, thus reinforcing its neuroprotective mechanism. Crucially, while pregnenolone effectively down-regulates pro-inflammatory responses dependent on MyD88, it does not appear to influence the MyD88-independent pathway of TLR4 signaling. This is evident from unaltered levels of LPS-induced IFN-β secretion in RAW264.7 cells [68]. 

Progesterone reduces pro-inflammatory TLR activation by decreasing the phosphorylation of the IκBα, thereby enhancing its expression. This leads to diminished phosphorylation of NF-κB and inhibits its expression and nuclear translocation. Progesterone also downregulates TLRs, MyD88 and CD14 levels and concomitant inflammatory cytokines while upregulating anti-inflammatory proteins such as suppressor of cytokine signaling (SOCS) 1 [69,71,72,73,294,307,308,309]. Furthermore, its structural similarities with pregnenolone and allopregnanolone suggest it may share the same abilities to inhibit TLR activation described above. Moreover, progesterone modulates the production of nitric oxide (NO) and IL-12 in macrophages through both glucocorticoid receptor-mediated and progesterone receptor-mediated mechanisms following TLR4 signal activation [70]. In the context of TBI, progesterone dampens the activation of TLR2 and TLR4 signaling pathways. This reduction in TLR2 and TLR4 levels, coupled with decreased NF-κB binding activity, leads to the suppression of inflammatory cytokine production, specifically IL-1β, TNF-α, and IL-6, in the pericontusional cortical region [74]. Furthermore, in BV-2 microglia, progesterone suppresses TLR4 activation induced by LPS and reduces the expression of proinflammatory mediators, including TNF-α, inducible NO synthase (iNOS), and cyclooxygenase-2 (COX-2) as well as the reduction of NO release [294,310]. In macrophages, progesterone decreases IL-6 and NO production following TLR4 and TLR9 signal activation [69] and suppresses the secretion of TNF-α and IL-6 in response to TLR4 and TLR2 signal activation [68]. It also inhibits LPS-induced iNOS expression, TLR4 expression, and NF-κB activation. Additionally, progesterone up-regulates the expression of SOCS1 protein as a negative feedback regulator [69]. Prior treatment of cells with progesterone as well as estradiol inhibits the activation of TLR2 and TLR4 pathways induced by TLR2 agonist Pam3CSK4 and TLR4 agonist LPS, resulting in reduced TNF and IL-6 levels in human cord blood mononuclear cells in newborns [309]. Moreover, progesterone and estradiol downregulate TLR2, CD14, and COX2, inhibit NF-κB signaling, and reduce prostaglandin E2 secretion in LPS-activated primary human monocytes, independently of MAPK pathway [308]. In cases of pre-eclampsia, progesterone inhibits TLR4 activation in human PBMCs. Increasing progesterone concentration correlates with reduced mRNA levels of TLR4, MyD88, and NF-κB, while elevating IκBα protein levels. Furthermore, progesterone reduces the expression of TNF-α and IL-6, underscoring its potential to modulate the immune response in this condition [71]. 

### 2.4. Mechanisms of Action of Androstane Neuroactive Steroids on Toll-like Receptors

Androstane neuroactive steroids like estrogens, androgens, DHEA, and ADIOL have complex effects on TLR expression and activation. These effects are notably context-dependent, influenced by specific diseases, and vary across different cell types [137]. 

ADIOL, also known as androstenediol or 5-androstenediol, holds promise as a potent anti-inflammatory agent, particularly within the brain where it can mitigate inflammatory responses triggered by TLR activation. ADIOL is synthesized from its precursor, DHEA, within microglia by reducing the 17-keto group [195]. It serves as a selective modulator of estrogen receptors (ER), effectively suppressing inflammatory reactions in microglia and astrocytes [108]. Studies by Salama et al. revealed that ADIOL reduces NF-kB levels in both the striatal and nigral regions of a rat model with rotenone-induced Parkinson’s disease (PD) [311]. Additionally, ADIOL promotes the production of the anti-inflammatory cytokines IL-4 and IFN-γ in experimental autoimmune encephalomyelitis, mitigating axonal damage resulting from demyelination by shifting microglial polarization toward a reparative state [312,313]. ADIOL also exhibits inhibitory effects on the expression of IL-1β when human microglia are stimulated with the TLR4 agonist LPS [314], underscoring its role in countering inflammation following TLR4 signal activation. Hanna et al.’s research further supports ADIOL’s anti-inflammatory potential by demonstrating reduced levels of TNF-α and IL-6 in the striatal area when administered before exposure to 3-nitropropionic acid-induced neurotoxicity [315]. Moreover, ADIOL has demonstrated the ability to suppress pro-inflammatory cytokines, including TNF-a, in murine models of carrageenan-induced pleurisy and septic shock resulting from LPS-induced TLR4 activation. It achieves this through its binding and transactivation of sex steroid receptors, with a preference for ERβ over ERα and androgen receptors (AR) [316]. ADIOL also effectively inhibits the TLR4 inflammatory pathway, reducing TLR4, NF-kB, and HMGB1 levels in peritoneal tissues [106]. 

Estrogens and androgens can display both anti-inflammatory and pro-inflammatory effects on TLRs, depending on the context. Estrogen-activated ERα in the brain reduces inflammatory mediator production and suppresses microglial activation via cytoplasmic PI3K induction. This activity inhibits the intracellular transport of NF-kB, which is initiated by the activation of TLR4 signaling through LPS stimulation [116,117,317]. Estradiol shortens the pro-inflammatory phase induced by LPS in mouse macrophage RAW 264.7 cells by activating intracellular ER. Estradiol, by modulating the SOCS3 and STAT3 signaling pathways, directs the inflammatory process towards the “acquired deactivation” phenotype. This phenotype, dependent on IL-10, is instrumental in tissue remodeling and the restoration of homeostasis [122]. However, estradiol, administered chronically, enhances peritoneal macrophage activation via ERα. This is achieved by downregulating the PI3K/protein kinase B (Akt) pathway, which, in turn, relieves its inhibitory effect on TLR4 signaling. Consequently, peritoneal macrophages become more responsive to TLR4 activation, resulting in increased proinflammatory cytokine production and iNOS expression [132,133]. 

Estradiol and progesterone had no significant effect on the expression of TLRs in human fallopian tube epithelial cells when administered separately. However, the co-administration of these neuroactive steroids led to significant changes in TLR expression [72]. Estradiol and progesterone both inhibit TLR3 signal activation and IL-6 production in these cells [131]. In the context of bone metabolism, estrogen downregulates the IL-6 gene by inhibiting NF-kB and potentially influencing CCAAT enhancer binding protein beta, even without a functional ER binding site [128]. 

The interaction among estradiol, TLR5, and the immune response in the bladder is intricate. Estradiol and progesterone both contribute to a reduction in TLR5 expression and impact its functional activity in the bladder, which is evident in the levels of IL-6 produced in response to flagellin. Notably, there is an inverse relationship between TLR5 expression and IL-6 production. Estradiol enhances IL-6 production, whereas progesterone intensifies it even further when compared to hormone-free or combined estrogen-progesterone environments [73]. Estradiol can also enhance TLR8 expression in human PBMCs. This process is ERα-dependent, involving direct DNA binding of ERα to an estrogen response element located downstream of the TLR8 gene [130]. 

In vitro, testosterone reduces TLR4 expression and sensitivity to TLR4-specific triggers in mouse macrophages. In vivo, the removal of endogenous testosterone increases susceptibility to endotoxic shock and elevates TLR4 expression in isolated mouse macrophages, suggesting a link between testosterone’s immunosuppressive effects and TLR4 regulation [135]. In contrast, dihydrotestosterone (DHT) increases TLR4 expression, while LPS boosts AR expression, promoting hepatocellular carcinoma progression and migration [318]. Additionally, testosterone persistently dysregulates hepatic expression of TLR6 and TLR8 induced by Plasmodium chabaudi malaria through epigenetics, while DHT modulates TLR7 and inhibits TLR9 expression by preventing cell apoptosis of plasmacytoid dendritic cells [136,319]. 

In BV-2 microglia, DHEA demonstrates effective inhibition of nitrite production induced by LPS-stimulated TLR4 activation, achieving this through a dose-dependent reduction in iNOS mRNA and protein levels, without necessitating new protein synthesis or mRNA destabilization. This highlights DHEA’s potent regulatory influence on microglial immune responses [320]. Similarly, in macrophage RAW 264.7 cells, DHEA attenuates inflammatory responses triggered by LPS-induced TLR4 activation. This suppression is accomplished by inhibiting Akt, MAPK, and the downstream NF-κB pathway while concurrently promoting the activation of autophagy-related nuclear factor erythroid 2–related factor 2 [112]. Furthermore, DHEA exhibits the potential to enhance neutrophil phagocytosis, reduce reactive oxygen species production, and decrease IL-8 release by modulating NF-κB signaling [134]. However, in a pig model of trauma and delayed sepsis, LPS-induced TLR4 signaling leads to a significant reduction in endogenous DHEA levels. Despite the administration of exogenous DHEA effectively elevating DHEA levels in treated animals, it fails to mitigate the subsequent systemic inflammatory response and organ dysfunction triggered by TLR4 activation, ultimately resulting in septic symptoms and pulmonary failure [321]. Moreover, DHEA administration in mice following sepsis induction resulted in the restoration of TLR expression, particularly TLR2 and TLR4 mRNA, in splenic macrophages. This reversal of TLR down-regulation is associated with a reduction in anti-inflammatory IL-10 responses and an increase in proinflammatory TNF-α production [138]. In summary, DHEA shows promise in modulating immune responses via TLR activation in microglial and macrophage cell lines. However, its effectiveness in an animal trauma and sepsis model is limited, and its impact on TLR expression presents complex outcomes. Additional research is required to gain a comprehensive understanding of DHEA’s role in immune modulation.

The interactions between androstane neuroactive steroids and TLRs reveal a complex scenario of immune regulation with implications for various physiological and pathological processes. Further research in this field holds promise for understanding and using these interactions for therapeutic purposes, particularly in the context of neuropsychiatric disorders. 

## 3. Benefits of Neurosteroid-Mediated Neuroimmune Modulation in Neuropsychiatric Disorders 

### 3.1. Depression

The association between depression and immune responses has been extensively documented in numerous studies, underscoring the pivotal role of inflammatory TLR signaling [152,153,257,322,323,324,325,326,327]. Proinflammatory cytokines and chemokines resulting from TLR activation are now recognized as potential biomarkers for depression [152,328], PPD [64,329,330], and PTSD [331,332,333].

In MDD, patients often exhibit elevated levels of TNF-α and IL-6, along with upregulated MyD88 and TRIF components [323,324,334]. TLR4 expression correlates with MDD symptoms, anxiety, and weight loss, and postmortem studies on MDD suicides reveal elevated TLR4 levels in the brain and blood mononuclear cells [335,336,337,338].

In the etiology of PPD, inflammation plays a significant role, with TLR4 and TLR7 pathway activation leading to increased pro-inflammatory markers such as TNF-α and IL-6 [64,258,339]. IL-6, IL-1β and TNF-α levels correlate positively with depressive scores in women with PPD, and elevated IL-1β concentrations are found in the CSF of patients developing persistent pain and/or PPD after cesarean delivery [64,340,341]. Elevated levels of IL-6 and TNF-α at delivery are associated with postpartum depressed mood, and increased IL-6 and its receptor levels are observed in women with PPD [342,343]. Additionally, mothers who deliver prematurely show increased IL-6 and IL-8 levels [344].

PTSD involves dysregulated HPA axis activity, resulting in elevated glucocorticoid levels and activation of microglia [345,346]. Glucocorticoid upregulation activates the inflammatory TLR2 pathway [347] and promotes the release of IL-1β, IL-6, and TNF-α from microglia [348,349,350,351]. Additionally, elevated glucocorticoids activate astrocytes, leading to excessive IL-1 release and disruption of glutamate homeostasis, potentially causing cellular degeneration [352]. Animal models and clinical studies have shown an association between elevated glutamate levels in the CSF and PTSD [353,354,355].

Preclinical research has shown that pregnenolone and/or allopregnanolone levels are reduced in animal models of stress, depression, and PTSD [39,40]. Furthermore, clinical investigations have confirmed these findings among individuals with depression [43,44,45] and those with a history of depression [46]. Given the limitations observed with these steroids, researchers have explored the potential therapeutic effects of neurosteroid administration. Allopregnanolone and its precursor molecules, such as pregnenolone/progesterone, have shown promising therapeutic efficacy in animal models exhibiting depression-like behavior induced by chronic stress [356]. Clinical studies consistently demonstrate the remarkable effectiveness of allopregnanolone in treating PPD [62,63,64,65,76,357,358].

In our recent study involving patients treated with brexanolone (a commercial formulation of allopregnanolone) for PPD, the therapeutic effects were associated with the inhibition of inflammatory mediator production and suppression of inflammatory responses to TLR4 and TLR7 pathway activators [64]. Brexanolone reduced baseline levels of the inflammatory markers TNF-α and IL-6, and these effects predicted improvement in the Hamilton Rating Scale for Depression (HAM-D) scores. Additionally, brexanolone treatment inhibited the blood cell response to the inflammatory immune activators LPS and imiquimod, suggesting its blockade of TLR4 and TLR7 pathways, activated by LPS and imiquimod, respectively. These responses also predicted improvement in HAM-D scores in the patients, representing a significant advancement in our understanding of brexanolone’s effectiveness and suggesting that inflammatory signaling may contribute to the etiology of PPD. This finding marks the first clinical correlation between allopregnanolone’s inhibition of inflammatory signaling and its clinical efficacy. Ongoing studies by our group and others are exploring the therapeutic potential of neuroactive steroids in various disorders, including depression. The emerging evidence underscores the significance of inhibiting neuroimmune signaling in their mechanisms of action against inflammatory conditions.

Moreover, in a double-blind phase 3 randomized clinical trial, zuranolone, also known as SAGE-217 (an allopregnanolone derivative), showed significant improvements in depressive symptoms, as measured by the HAM-D score, among women with PPD. Sustained differences favoring zuranolone were noted from day 3 to day 45, accompanied by positive outcomes in response and remission rates, along with improvements in anxiety symptoms [359]. Additionally, in a double-blind phase 2 trial, administration of zuranolone daily for 14 days to patients with MDD resulted in a reduction in depressive symptoms on day 15. Adverse events were more common in the zuranolone group than in the placebo group. Further trials are needed to determine the durability and safety of zuranolone in MDD and to compare zuranolone with available treatments [360].

However, the study investigating ganaxolone, another synthetic derivative of allopregnanolone, for the treatment of PTSD did not demonstrate significant differences between ganaxolone and placebo in improving PTSD symptoms. However, challenges with dosing and pharmacokinetics may have contributed to these findings. Future research on ganaxolone should consider higher dosing, rigorous dosing adherence monitoring, longer placebo-controlled testing, and targeting treatment to specific PTSD subpopulations with dysregulated neuroactive steroid levels [361].

Pregnenolone, as an add-on therapy, shows promise in alleviating depressive symptoms in adults with bipolar disorder (BPD), highlighting its potential as a safe and effective treatment option for BPD-associated depression [43]. Testosterone, as observed in randomized placebo-controlled clinical trials, is associated with a significant reduction in depressive symptoms among men, demonstrating both effectiveness and efficacy [362].

Interestingly, the competitive 3β-hydroxysteroid dehydrogenase (3β-HSD) inhibitor, trilostane, modulates the levels of neuroactive steroids in both peripheral and brain regions. Notably, trilostane demonstrates facilitative effects on the antidepressant activity of DHEAS [363,364].

In summary, neurosteroid-mediated neuroimmune modulation presents a promising avenue for treating neuropsychiatric disorders like depression. Studies highlight the role of inflammatory signaling in these conditions, with drugs like brexanolone showing effectiveness in alleviating symptoms, particularly in PPD. While challenges persist, ongoing research underscores the potential of neuroactive steroids to offer novel therapeutic options for these conditions, complementing existing treatment strategies.

### 3.2. Substance Use Disorders

The influence of alcohol and psychoactive drugs such as cocaine, methamphetamine, cannabis, and nicotine on neuroinflammation and immune responses is a growing area of research in both clinical and preclinical fields. Understanding their impact on inflammatory pathways and neuroimmune function is pivotal for unraveling their neurobiological mechanisms and identifying potential therapeutic interventions, including the promising utilization of neurosteroids.

Elevated levels of MCP-1 have consistently been observed in various brain regions, including the ventral tegmental area, substantia nigra, hippocampus, and amygdala, among individuals with AUD [365], with similar findings in mouse brains [366]. Moreover, alcohol exposure triggers the release of pro-inflammatory cytokines and activates chemokine/cytokine receptors in glial cells, such as astrocytes and microglia [266,367,368,369].

Excessive neuroinflammation associated with alcohol addiction is often attributed to the activation of inflammatory TLR pathways. Recent research highlights the involvement of the HMGB1–TLR–MyD88–NF-κB signaling pathway in contributing to neurodegeneration observed in the orbitofrontal cortex of postmortem human subjects with AUD [160]. Chronic and acute alcohol exposure activates various TLR-mediated pathways, leading to microglia activation and elevated levels of pro-inflammatory cytokines and chemokines in the brain [147,158,159,370,371,372,373,374,375,376,377,378]. Alcohol also disrupts the homeostatic balance of pro-inflammatory and anti-inflammatory factors in the brain, favoring pro-inflammatory factors [146].

Chronic cocaine use has been associated with increased levels of IL-6 and decreased levels of IL-10, as well as an imbalance in pro-inflammatory and anti-inflammatory markers. Additionally, cocaine exposure influences glutamate release and transporters, contributing to neuroinflammation and microglial activation [58,379,380,381].

Methamphetamine exposure exacerbates oxidative stress, apoptosis, and neuroinflammation, characterized by microglial activation and the release of inflammatory cytokines. Methamphetamine-induced dopamine and glutamate release further exacerbate neuroinflammation [382,383,384,385,386,387].

Cannabis exposure exhibits immunomodulatory effects, but some data suggest a pro-inflammatory impact, particularly in individuals with cannabis use disorders [305,388,389,390]. 

Nicotine’s effects on the immune system are complex and vary depending on exposure and context, with evidence suggesting both pro-inflammatory and anti-inflammatory effects [147,391,392,393,394].

These dysregulations in neuroimmune and immune signaling induced by substance abuse can compromise neural functions, lead to neurodegeneration, and increase neurotoxicity, all contributing to the behavioral manifestations associated with substance use disorders.

Preclinical and clinical investigations underscore the beneficial impact of neuroactive steroid treatment on substance abuse outcomes. Milivojevic et al. (2023) [61] found that pregnenolone treatment in individuals with AUD significantly reduces stress and alcohol cue-induced craving, while also alleviating stress-induced anxiety and normalizing HPA axis and autonomic responses. Additionally, studies in alcohol-preferring P rats by O’Dell et al. (2005) [395] and Besheer et al. (2010) [48] demonstrate that systemic administration of pregnenolone, epiallopregnanolone (3β,5β-THP), or the synthetic neurosteroid 3α,5β-20-oxo-pregnane-3-carboxylic acid (3α,5β-PC) reduces ethanol self-administration. Furthermore, Ornelas et al. (2023) [50] found that infusion of allopregnanolone in the nucleus accumbens decreases alcohol self-administration in female P rats.

It should be noted that P rats represent a model of innate TLR activation where the effects on brain TLR pathways can be examined in the absence of peripheral immune activation. Overactivation of inflammatory TLR signaling pathways leads to an imbalance in pro-inflammatory and anti-inflammatory factors favoring the former [146]. Allopregnanolone treatment of P rats inhibits inflammatory MyD88-dependent TLR4 and TLR7 signaling, resulting in reduced MCP-1 levels in the brain. Additionally, allopregnanolone enhances anti-inflammatory TRIF-dependent TLR4 signaling, leading to increased IL-10 levels in the brain [36,67,80].

In individuals with cocaine use disorder (CUD), after progesterone treatment, both men and women experienced reduced cue-induced cocaine craving and cortisol responses, along with improved inhibitory control performance. However, women reported lower negative emotions and higher relaxed mood ratings after stress exposure, unlike men [58]. Additionally, administering progesterone to individuals with CUD leads to heightened levels of GABAergic neurosteroids, namely allopregnanolone and pregnanolone, both in men and women. Notably, high allopregnanolone levels were associated with normalized cortisol responses to stress, improved mood, enhanced cognitive performance, and reduced cocaine craving. However, reduced levels of pregnenolone and androstanediol were associated with prolonged durations of cocaine use [59,60]. Pregnenolone supplementation significantly increases pregnenolone levels and effectively reduces stress and cocaine cue-induced craving and anxiety in individuals with CUD. Additionally, it demonstrates a notable reduction in stress-induced autonomic arousal [226].

Additionally, pregnenolone has emerged as a potent negative allosteric modulator of the CB1 receptor, effectively counteracting the effects of Δ9-tetrahydrocannabinol (THC), the primary active compound in Cannabis sativa (marijuana). This suggests a potential protective role against cannabis intoxication. Contrary to its conventional classification solely as a precursor to neurosteroids, evidence indicates that pregnenolone, rather than its downstream neurosteroids, mediates this inhibition of THC effects via CB1 receptors. Notably, THC administration significantly boosts pregnenolone synthesis in the brain through CB1 receptor activation, initiating a negative feedback loop where pregnenolone acts as a protective mechanism against CB1 receptor overactivation [202].

Importantly, AEF0117, also known as 3β-(4-methoxybenzyloxy)pregn-5-en-20-one, was engineered to be an unmetabolized derivative of pregnenolone, targeting selective inhibition of THC effects via the CB1 receptor. Preclinical studies in animals demonstrated reduced cannabinoid self-administration and THC-related impairment without significant adverse effects following AEF0117 treatment. Phase 1 trials in healthy volunteers confirmed the safety and tolerability of AEF0117, while phase 2a trials in individuals with cannabis use disorder showed significant reductions in cannabis subjective effects and self-administration without precipitating withdrawal. These findings suggest that AEF0117 could be a safe and effective treatment for cannabis use disorder [66].

Moreover, it is known that CB1 receptors can interact with TLR receptors, implying that inhibition of CB1 receptors by pregnenolone or its derivatives could also mitigate inflammatory TLR signaling [305,306].

Although the studies did not directly assess inflammation, the known anti-inflammatory properties of progesterone, allopregnanolone and pregnenolone suggest a potential area for future research in investigating their impact on inflammation in cocaine- and cannabis-dependent individuals. Moreover, further research is warranted to assess the effects of neurosteroid therapy on other substance use disorders where the field currently lacks sufficient information, such as nicotine and methamphetamine.

In summary, substance abuse, including alcohol and psychoactive drugs, can disrupt neuroimmune signaling, leading to neurodegeneration and behavioral issues in substance use disorders. Neuroactive steroid treatments, like allopregnanolone and pregnenolone, show promise in alleviating craving, anxiety, and stress-induced responses and inhibiting pro-inflammatory signaling while enhancing anti-inflammatory signaling. Future research should explore their potential anti-inflammatory effects and therapeutic applications across various substance use disorders.

### 3.3. Pain and Neurological Injuries

Neurological injuries, acute pain, chronic pain, migraines, neuropathic pain, and allodynia are associated with activation of both the adaptive and innate immune systems [182,184]. TLRs, particularly TLR4, regulate pain perception and prolongation through the expression of cytokines [396,397,398]. Sex-specific differences in pain perception have highlighted the necessity for tailored treatments [399,400]. Notably, sexual specificity is observed in pain related TLR signaling, where TLR4 inhibition directly modulates pain perception in males but not females [401,402].

Allodynia has been reversed in transgenic mice lacking TLR2, TLR3, TLR4, TLR5, or MyD88 genes, with MyD88-dependent pathway activation identified as the primary mediator of allodynia [401,403]. Although not specifically employing neuroactive steroids, the observed analgesic effect is attributed to the inhibition of the TLR–MyD88 pathways, like the mechanisms inhibited by allopregnanolone, pregnenolone, and progesterone [36,37,67,71].

Studies in rats have shown that progesterone treatment after spinal nerve injury led to a decrease in pain behavior and allodynia, along with reduced mRNA levels of IL-1β, IL-6, and TNF-α [404,405]. Allopregnanolone and progesterone have also demonstrated a decrease in acute NF-kB, IL-6, IL-1β, and TNF-α expression in TBI rat models [52,406,407,408]. Additionally, in ovariectomized female rats, estrogen and progesterone have been shown to reduce edema and cytokine expression observed in TBI [409,410].

Testosterone has been found to decrease pro-inflammatory cytokines and increase the anti-inflammatory cytokine IL-10 in males, providing protection against muscle pain in both females and males [411,412,413].

Emerging evidence from male and female Iraq/Afghanistan-era veterans suggests that decreased neurosteroid levels are associated with increased pain and TBI symptoms, indicating a potential role for neurosteroids as biomarkers [414,415,416].

Importantly, in the randomized, double-blind, placebo-controlled clinical trial involving male Iraq- and Afghanistan-era US military veterans with chronic low back pain, adjunctive treatment with pregnenolone showed significant promise. Participants who received pregnenolone reported a substantial reduction in pain intensity ratings after 4 weeks of treatment compared with those who received placebo. Additionally, pregnenolone treatment led to improvements in pain interference scores for work and activity. These findings suggest that pregnenolone may serve as a safe and effective adjunctive treatment for male veterans with chronic low back pain [417]. Further research and exploration into the long-term effects and optimal dosing of pregnenolone are warranted to fully elucidate its therapeutic potential in pain management.

In summary, pain and neurological injuries are closely tied to immune system activation, notably through TLR4. Sex-specific differences underscore the need for personalized, tailored treatments. Neuroactive steroids and hormones such as estrogen and testosterone offer promising avenues for pain management in neurological injuries.

### 3.4. Seizure Disorders

Seizures often coincide with several other disorders, including migraine with aura, alcohol dependence, pain, depression, and various neurological and psychological conditions [418,419,420,421]. Given the potentially life-threatening nature of seizures, much research has been directed towards managing seizure episodes rather than addressing the underlying disorders themselves. Consequently, numerous seizure models and treatments have been developed, primarily focusing on regulating neuronal signaling. GABAergic neuroactive steroids like allopregnanolone are proposed as anti-convulsants, while sulfite active steroids such as PS and DHEAS are known to have convulsant and epileptogenic properties [422,423].

The depletion and lower levels of neurosteroids have been implicated in seizures, as observed in conditions such as catamenial epilepsy, where seizures are more frequent during periods of low progesterone levels [424,425]. Treatments involving progesterone have shown promise in reducing seizure frequency in women with intractable catamenial epilepsy [426], with its anti-convulsant effects suggested to be mediated by metabolites such as 3α,5α-THDOC and primarily allopregnanolone [427,428,429,430].

These findings have led to clinical studies involving the exogenous β-methylated analog of allopregnanolone as an anti-convulsant. A recent meta-analysis by Meng et al. (2023) [431] reported the efficacy of ganaxolone in reducing seizure frequency by up to 50% for refractory seizures, leading to its FDA approval in 2022 for the treatment of seizures associated with cyclin-dependent kinase-like 5 (CDD) deficiency disorder [432]. In the open-label extension of the Marigold study (NCT03572933), ganaxolone showed sustained reductions in major motor seizure frequency over 2 years in patients with CDD. Most patients experienced significant improvements, with safety findings consistent with earlier phases, supporting ganaxolone’s efficacy and safety profile for CDD-associated seizures [433].

Interestingly, trilostane, a potent inhibitor of 3β-HSD, has recently been found to significantly increase levels of various neurosteroids, particularly allopregnanolone, both in the brain and peripherally. Notably, in the kainic acid model of temporal lobe epilepsy, trilostane treatment has demonstrated a remarkable ability to slow down epileptogenesis, resulting in a significant reduction in seizure occurrence compared with the control group receiving vehicle. These findings suggest that the trilostane-induced elevation of neurosteroids may possess a disease-modifying effect in epileptic brains [434,435]. 

While extensive research has explored the influence of neuroactive steroids on epileptogenesis, seizure frequency, and their modulation of neuronal currents via GABAergic signaling, emerging evidence indicates that the anti-inflammatory properties of neuroactive steroids could amplify their therapeutic potential for seizure management.

Mounting evidence underscores the pivotal role of immune and inflammatory processes in the onset and progression of seizures. Various triggers, including infection, febrile seizures, neurotrauma, and stroke, induce innate immune mechanisms and subsequent inflammatory responses in the brain, leading to acute symptomatic seizures and an increased risk of epilepsy [185,186,271,273,436]. Immune and glial cells, notably astrocytes and microglia, are central to these processes. During status epilepticus, activated astrocytes disrupt synaptic equilibrium, exacerbate excitotoxicity, and contribute to seizure occurrence [437,438]. Notably, stimulating the astrocytic inflammatory TLR4–MyD88–ERK pathway during early development induces excitatory synaptogenesis and increases susceptibility to seizures [439,440]. Microglia exhibit remarkable plasticity in transcription, morphology, and function, which dynamically evolve throughout the course of epilepsy [441]. Inhibiting the activation of astrocytic and microglial cells holds promise as a treatment strategy, necessitating the development of novel approaches with anti-inflammatory effects. These approaches aim to address the underlying inflammatory processes contributing to epilepsy [442,443].

Advancements in understanding the molecular intricacies of the innate immune system, particularly TLR signaling, have unveiled its significant contribution to seizure activity. Activation of TLR signaling results in increased production of various inflammatory modulators [187,271,272,444,445,446,447], along with rapid post-translational changes in ion channels that enhance excitability and transcriptional alterations in genes associated with neurotransmission and synaptic plasticity, ultimately reducing seizure thresholds chronically [186,448]. Experimental studies have demonstrated the critical role of TLR4 in seizure initiation and propagation, with its ligand HMGB1 emerging as a potential therapeutic target [440,449]. HMGB1, a member of the damage-associated molecular patterns family, is upregulated in experimental epilepsy models and interacts with TLR4 receptors, contributing to epilepsy pathophysiology [272]. Studies on TLR knockout mice and expression levels in epileptic patients further emphasize the involvement of TLR-dependent pathways in seizure development [274,450,451,452].

Recent studies have shown that allopregnanolone inhibits inflammatory MyD88-dependent TLR pathways, resulting in decreased production of MCP-1, TNF-α, IL-1β, IL-6, and HMGB1, and enhances the TRIF-dependent anti-inflammatory TLR4 pathway, resulting in increased production of IL-10 and BDNF in the brains of P rats. Allopregnanolone has been found to have similar effects in mouse and human macrophages [36,37,67,80]. This innovative finding highlights the potential of neurosteroids to influence the neuroimmune system through both MyD88-dependent and TRIF-dependent TLR pathways in epilepsy and a wide variety of conditions involving inflammation.

### 3.5. Neurodegenerative Diseases 

Neuroinflammation is one of the hallmarks of the pathology of neurodegenerative disorders, and some evidence suggests a role of TLRs in mediating this inflammatory response. Parkinson’s disease (PD) is a progressive neurodegenerative disorder that is characterized by the deterioration of dopaminergic neurons, leading to motor deficits such as tremors, bradykinesia, and rigidity [453]. Several studies have demonstrated TLR2 upregulation in both blood and brain postmortem tissue of PD patients, with enriched expression observed in neurons and microglia within the brain [454,455]. Other evidence reports increased proinflammatory cytokines, including TNF-α, IL-1β, IL-2, IL-4, and IL-6, in blood, brain, and CSF of PD patients [455,456]. Furthermore, as the prevalence of PD is nearly two times higher in men than in women [457,458,459], it has led to the hypothesis that female sex hormones might play a protective role against the factors contributing to PD pathology [460,461]. In line with this, several neurosteroids have been implicated as potentially beneficial therapeutics for the treatment of PD. 

First, allopregnanolone and 5α-dihydroprogesterone, but not progesterone, levels in plasma and CSF are downregulated in PD patients relative to healthy individuals [462]. Allopregnanolone also restored degraded TH-immunoreactive neurons in the nigrostriatal tract and improved balance and motor coordination in a PD model mouse (1-methyl-4-phenyl-1,2,3,6-tetrahydropyridine lesion) [463]. As a further example, allopregnanolone improved motor impairments and reduced COX-2 levels in a 6-hydroxydopamine (6-OHDA) model rat [464]. Progesterone, a precursor to allopregnanolone, was shown to be neuroprotective against 6-OHDA and 1-methyl-4-phenylpyridinium-induced cell death in an SH-SY5Y neuronal cell line [465]. Furthermore, progesterone reversed the loss of striatal dopamine, dopamine and vesicular monoamine transporters and prevented the loss of BDNF and the increase of GFAP [466]. As for androstane steroids, DHEA treatment rescued depleted TH-positive neurons and recovered ERK phosphorylation [467]. ADIOL ameliorated rotenone-induced elevation of NF-κB and expression of iNOS and IL-6 [311]. Taken together, these studies suggest a potential protective role of pregnane and androstane steroids in PD pathology, in part, through neuroimmune regulation. 

Alzheimer’s disease (AD) is a progressive neurodegenerative disorder characterized by the deterioration of cognition and memory and associated with the accumulation of amyloid-beta and tau proteins [468]. It is well-known that neuroinflammation contributes to the pathogenesis of AD. Specifically, microglial activation is induced by the presence of amyloid-beta plaques as an attempt to phagocytose and clear these plaques [469,470]. Proinflammatory signaling molecules, such as TNF-α, IL-1β, and IL-6, play central roles in AD pathology [471,472]. TNF-α and IL-1β levels have been reported to be enriched in the brains and blood of AD patients [473,474,475]. The release of these cytokines by activated microglia contributes to the amplification of neuroinflammation and exacerbates synaptic dysfunction [476]. Moreover, the presence of inflammatory signaling molecules is identified as a risk factor for the development of AD [477,478]. As neurosteroids have been shown to confer therapeutic benefit in the context of neuroinflammatory mediation, there is evidence to suggest they may be a treatment option for AD. 

In fact, in preclinical rodent studies, allopregnanolone reduced amyloid-beta accumulation, increased the survival of neural progenitors, restored memory and learning performance, and decreased microglial activation in an AD-model mouse [479,480,481]. In clinical studies, allopregnanolone is currently being investigated in a phase II trial as a potential regenerative therapy in AD patients, with a particular focus on assessing hippocampal volume and cognition (NCT04838301) [81]. Preliminary results from a phase I trial suggest that allopregnanolone is well tolerated and safe in patients with AD [482]. As the reduction in estrogen in postmenopausal women is a risk factor for the development of AD, it raises the question of whether female sex hormones may be protective against AD pathogenesis [483,484]. Indeed, AD depletion of estrogen and progesterone via ovariectomy in rodents results in worsening of AD-like pathology, but estrogen treatment prevents this worsening. However, progesterone treatment has contrasting effects as it does not ameliorate amyloid-beta accumulation but does reduce tau phosphorylation [485]. The protective role of female sex hormones is well-documented, and their potential therapeutic benefit in AD remains to be further clarified. Other neurosteroids have been implicated in AD, as DHEAS concentrations are found to be lower in AD patients relative to control, whereas DHEA levels are not different [486,487]. In fact, DHEA treatment did not improve cognitive performance in a clinical study with patients with AD [488]. Interestingly, it was found that high DHEAS levels were associated with faster cognitive decline, and the ratio of cortisol/DHEAS is positively correlated with tau accumulation [489]. This apparent contradiction reflects the complex relationship between different neurosteroids with distinct actions and AD pathology. Finally, investigations into the therapeutic potential of 3α,5α-THDOC for AD have found that 3α,5α-THDOC reduces amyloid-plaque accumulation and size in an AD model rat [490]. Taken together, these studies highlight the potential for some neurosteroids to regulate neuroimmune and inflammatory processes, holding promise for their broader application in the treatment of neurodegenerative disorders.

### 3.6. Neurodevelopmental Disorders and Autism Spectrum Disorder (ASD)

Neurodevelopmental disorders, including autism spectrum disorder (ASD), result from a complex interplay of genetic, mental, and environmental factors [491]. Prenatal exposures such as stress, drugs, and viruses, along with preterm birth and childhood experiences, can significantly impact the immune system. Notably, during pregnancy, elevated levels of estrogen and progesterone stimulate the expression of anti-inflammatory cytokines such as transforming growth factor (TGF)-β, IL-4, and IL-10 [492]. Research in rats indicates that the fetal immune state closely resembles that of the mother [493], and neuroactive steroids are transferred from the mother’s placenta to the fetus and also synthesized within the fetal organism [494]. This phenomenon explains the increased concentrations of progesterone and allopregnanolone following birth compared with the fetal phase [495]. Elevated levels of allopregnanolone, progesterone, IL-6, and IL-10 at birth have been associated with issues such as poor myelination, low birth weight, increased mortality, and encephalopathy among preterm newborns [495,496]. Subcutaneous administration of ganaxolone, a β-methylated analog of allopregnanolone, in preterm guinea pigs provided protection against the loss of myelination, hyperactive behavior, and premature mortality observed in untreated preterm control animals [497], highlighting the neuroprotective potential of allopregnanolone in mitigating neurodevelopmental disorders associated with preterm births.

ASD is a multifaceted neurodevelopmental condition influenced by genetic, environmental, and immune factors. Recent research suggests immune dysregulation may contribute significantly to its etiology. Elevated cytokine levels have been observed in both the peripheral and nervous systems of individuals with ASD [498], indicating potential immune involvement. Furthermore, studies have found heightened cytokine levels in brain tissue and spinal fluid of ASD patients [499,500]. Chew and Peers (2021) [501] proposed that disrupted neurosteroid production, particularly low levels of allopregnanolone, could contribute to immune and neurodevelopmental dysfunction in ASD. Clinical trials have shown that the neurosteroid pregnenolone can improve irritability, stereotypy, and hyperactivity in adolescents with ASD [502]. However, the impact of these steroids on the immune system remains understudied. Given the promising outcomes of neuroactive steroid treatments in mitigating ASD symptoms associated with impaired immune activation, further investigation into their regulatory role in the immune system is warranted.

## 4. Limitations and Challenges

### 4.1. Constraints and Potential Side Effects of Neurosteroid Therapy

The therapeutic use of neuroactive steroids has emerged as a promising avenue for the modulation of neuroimmune responses, offering a novel approach to address various neurological and psychiatric disorders. However, like any therapeutic intervention, the use of neurosteroids is not without its constraints and potential side effects. Understanding these limitations is crucial for the development and optimization of neurosteroid-based treatments.

For many years, therapy with allopregnanolone and other neuroactive steroids has raised greater caution by the scientific community given their positive modulation of GABA_A_ receptors. First, neurosteroid therapy increases endogenous neuroactive steroid levels, by increasing the substrates for steroidogenesis. This could lead to the production of other neurosteroids, with the same positive action on the GABAergic system or the opposite effects. For example, it has been demonstrated that administration of pregnenolone in rats induces the production of the GABAergic positive modulator, pregnanolone (3α,5β-3-hydroxypregnan-20-one), normally undetectable in rodents [503].

Second, it is well known that social drugs such as alcohol and nicotine can increase the levels of GABAergic neurosteroids [504,505,506]. The increase in these neurosteroids may interact with alcohol and nicotine, leading to deleterious effects that can affect important abilities such as driving or operating machinery. Moreover, several studies have shown that neuroactive steroids may increase alcohol reinforcement, consumption, and reinstatement of drinking in animal models of alcohol addiction, depending on the dose [48,507,508,509,510]. A recent study also unveiled a possible deleterious interaction between allopregnanolone administration and opioid system activation during forced swim stress in rats [511]. This result suggests the need for further studies to address potential limitations of allopregnanolone for psychiatric disorders involving HPA axis activation.

Moreover, allopregnanolone has been shown to promote cell proliferation in a human glioblastoma (GB) U87 multiforme cell line and the expression of genes associated with tumor progression, including TGF-β1, epidermal growth factor receptor, vascular endothelial growth factor, and cylin-D1 [512]. Additionally, allopregnanolone was shown to increase the migration and invasion of GB cells, potentially through the activation of the cellular proto-oncogene tyrosine-protein kinase Src pathway [513]. These results are further supported by the fact that sex steroid hormones, including estrogens and progestins, can induce the progression of GBs [514,515]. In the IGROV-1 ovarian cancer cell line, allopregnanolone increased cell proliferation, Ki67 expression, and cell migration [516]. Importantly, the primary literature surrounding the relationship between neurosteroids and cancer is very mixed, as allopregnanolone activation of membrane progesterone receptors (mPRs) decreased starvation-induced cell death and apoptosis in mPRδ-transfected cells and neuronal cells, suggesting a possible protective role [517]. These findings raise concerns about the potential role of allopregnanolone in promoting tumor progression; however, further research is needed to fully understand these implications and determine whether allopregnanolone may contribute to cancer development or progression in vivo. Additionally, it should be noted that the role of neurosteroids in cancer is likely dependent on various factors such as the type of cancer and the specific cellular context. 

Another concern is that therapy with allopregnanolone and other neuroactive steroids may lead to untoward effects, such as dependence and/or addiction, especially in patients with deficient GABAergic transmission, as seen in individuals with AUDs. Considering that therapy with neuroactive steroids may be a lifelong treatment for some patients, this possibility needs to be considered, and more studies need to be conducted to evaluate the long-term effects of neurosteroid therapy. In fact, it is well known that progesterone withdrawal can lead to anxiety and GABA_A_ receptor dysregulation in animals [518,519,520,521], suggesting that rapid allopregnanolone withdrawal may occur as well in human subjects. Potential side effects, tolerance development, and abuse potential of some neurosteroids are crucial aspects that remain to be fully clarified, and ongoing research is actively contributing to our evolving understanding of these factors.

As some neurosteroids are modulators of GABA_A_ receptors, a common side effect of neurosteroid treatment is sedation. In fact, patients receiving brexanolone therapy have reported fatigue, dizziness, sleepiness, dry mouth, flushes, and loss of consciousness [522]. Tolerance is known to develop after long-term exposure to GABA_A_ receptor agonists [523,524]. Indeed, allopregnanolone has been shown to induce both acute and chronic tolerance by decreasing the expression of GABA_A_ receptor subunits [525,526,527]. This underscores the potential for neurosteroids to change GABA_A_ receptor sensitivity, necessitating careful management to mitigate potential tolerance and maintain treatment efficacy.

At present, neurosteroids hold FDA approval solely for acute therapeutic interventions, mitigating concerns regarding tolerance and dependence. Brexanolone, for instance, is approved for a 60 h infusion [528], and zuranolone is approved as a 14-day oral medication [529]. However, in instances where chronic administration of neurosteroids might be necessary, close monitoring may be warranted regarding the potential development of tolerance and/or dependence. 

In summary, short-term neurosteroid therapy shows promise in modulating neuroimmune responses for neurological and psychiatric disorders, yet its chronic use likely entails constraints and potential side effects. Increased neurosteroid levels might lead to unpredictable effects, including interactions with social drugs like alcohol or nicotine. Additionally, concerns arise regarding the long-term effects and tolerance development associated with neurosteroid treatment. Ongoing research is essential to address these concerns and optimize the safety and efficacy of neurosteroid-based therapies. 

### 4.2. Ethical and Regulatory Considerations for Neuropsychiatric Treatment 

An estimated 22.8% of adults in the United States present with a mental, behavioral, or emotional disorder. While current treatment options exist, they are often ineffective in addressing the complex and heterogeneous nature of these disorders. As an example, for patients with MDD, only a third of patients experience significant symptom improvement with conventional therapies [530,531]. Thus, there is an ever-growing interest in novel therapeutics and emerging therapies for the treatment of psychiatric disorders. This includes the investigation of neurosteroids, which show immense promise for neuropsychiatric treatment.

Drug development is a high-cost, high-risk, and long process that has a staggering failure rate of 90% [532,533]. The estimated average cost of drug development ranges from USD 1.349 billion to USD 1.706 billion [534] and takes, on average, 10–15 years until approval [532]. Notably, neurological and psychiatric drugs face particularly daunting odds in gaining approval, with phase I clinical trials showing approval rates of 8.4% and 6.2%, respectively [535]. However, the challenges peak in phase II, where most clinical trials fail with an approximate 70% failure rate attributed to drug efficacy issues or significant off-target effects in patients [536,537,538]. Given these regulatory barriers, it is unsurprising that the number of drugs on the market and in phase III for psychiatric indications is low, and there exists a critical need for neuropsychiatric therapeutics. For psychiatric disorders in particular, the high failure rate is due, in part, to poor understanding and complexity of the pathophysiology of these disorders. Animal models, while invaluable, can have poor translational value for drug discovery research due to neuroanatomical differences and the limitations of behavioral assays [539]. Off-target effects and individual differences may also contribute to the challenges of predicting drug responses in clinical populations. The heterogeneity in presentation of psychiatric conditions and patient response to treatments can contribute to the apparent failures to meet specific endpoints in clinical trials [540]. Metrics for assessing symptom improvement in patients are often not sensitive enough to parse out specific benefits and can obscure the nuanced impact of treatments on various facets of psychiatric health. 

Even after FDA approval of a drug, barriers to patient access to treatment arise as the financial cost of novel therapeutics is often significantly higher than that of traditional therapies, raising ethical concerns. For example, the introduction of novel neuropsychiatric treatments, such as brexanolone (a formulation of the neurosteroid allopregnanolone) for PPD, comes with a substantial cost of approximately USD 34,000 [541]. This financial burden is further compounded by the additional cost of hospitalization during the required 60 h infusion period [522]. However, brexanolone has demonstrated remarkable efficacy in alleviating PPD symptoms, outperforming a placebo group based on HAM-D scores [62,358]. The accessibility of brexanolone treatment is further hindered by logistical challenges. Prolonged hospitalization during infusion can pose challenges for individuals who may face difficulties in managing the associated disruptions to life, work commitments, and childcare responsibilities. To address this challenge, there is a new oral formulation of brexanolone, zuranolone, introduced as an alternative. However, the financial strain remains substantial even with this option as it is priced at USD 15,900 without insurance [542]. Given the high efficacy of these treatments, one might suggest that the economic and personal burden incurred by postpartum depression through inability to work, etc., might be higher than treatment with brexanolone. Patients and physicians must choose between the acute, high cost associated with potentially efficacious and rapid symptom alleviation or opt for the comparatively lower cost of traditional treatments that may be less efficacious and administered long-term. As low socioeconomic status is a risk factor for PPD [543], it is important to recognize that these high treatment costs can perpetuate health disparities. This situation underscores a pressing ethical concern regarding equitable access to healthcare. 

## 5. Future Directions in Neurosteroid Research

The complexities of both systemic and neuroimmune signaling in neuropsychiatric diseases have brought attention to the emerging field of neurosteroid treatment research. The pharmacological properties of neuroactive steroids show promising applications due to their modulatory effects on a wide array of mechanisms. Within this review, we have examined previous findings and discussed the implications of neuroimmune modulation orchestrated by endogenous neurosteroids. However, many new avenues are yet to be explored, offering opportunities for innovation and discovery in the neurosteroid field.

Using traditional techniques such as co-immunoprecipitation, western blotting, and ELISAs, significant progress has been achieved in understanding the impact of neurosteroids on inflammatory TLR signaling pathways. For example, pregnane neurosteroids such as allopregnanolone and pregnenolone have demonstrated inhibitory effects on the binding of TLR2, TLR4, and TLR7 with MyD88. Additionally, they inhibit the binding of TLR4 with MD2 or the α2 subunit of GABA_A_ receptors. This understanding has provided valuable insights into the downstream effects on inflammatory-driven proteins, leading to the reduction of inflammatory chemokines and cytokines [36,37,67]. As previously noted, brexanolone, an intravenous formulation of allopregnanolone, has shown effectiveness in inhibiting the production of TNF-α, IL-1β, and IL-6 induced by the TLR4 agonist LPS and the TLR7 agonist imiquimod. This inhibition serves as an indicator of TLR4 and TLR7 signaling pathway suppression [64]. 

However, the exact molecular mechanisms and binding modalities behind these events have not yet been fully determined. Integrating complementary approaches such as computational modeling and molecular docking [544,545], surface plasmon resonance [546,547], cryo-electron microscopy [548,549], along with site-directed mutagenesis of target proteins [550], can provide a comprehensive understanding of these mechanisms. This integration paves the way for the development of novel therapeutic strategies targeting inflammatory signaling pathways.

MyD88 serves not only as a signaling protein in TLR pathways but also plays a crucial role in IL-1 receptor (IL-1R) signaling, which constitutes another significant neuroimmune signaling cascade. Presently, there are ongoing drug discovery endeavors concentrating on IL-1R pathways and related proteins like MyD88. This provides a solid foundation for advancing neurosteroid research within neuroimmune signaling [551,552,553]. Once these pathways are thoroughly understood, it opens the door to innovative avenues such as repurposing neurosteroids, exploring new synthetic analogs, and conducting ligand- or structure-based virtual screenings of neurosteroid-protein binding sites. Such initiatives hold the potential to uncover more effective and beneficial compounds for therapeutic use.

Despite their advantageous properties in alleviating neuroimmune signaling and GABAergic and other neurotransmission system modulation, the pharmacological and physicochemical properties of neurosteroids contain room for improvement for treatment purposes. The structural requirements for the anti-inflammatory properties of the pregnane neurosteroids have not been fully delineated but appear to involve the integrity of the D ring structure of pregnenolone, progesterone and allopregnanolone. Modifications of the D ring at C21 appear to reduce TLR inhibition in mouse and human macrophages as well as rat brain [36,37,67], but this modification does not impair increases in the anti-inflammatory modulators IL-10 and fractalkine observed in P rat brains [80,302]. The structural prerequisites for neurosteroid modulation of GABA_A_ receptors involve a hydrogen bond-donating 3α-hydroxy group on the steroid A-ring and a hydrogen bond-accepting group on the D ring, either at C20 of the pregnane steroid side chain or at C17 of the androstane ring [9,25,215]. Additionally, the orientation of the C5 hydrogen group is crucial for increased potency, though less so for activity [9,215]. Although advantageous, endogenous neurosteroids have been shown to have poor aqueous solubility, low bioavailability, quick metabolic turnover, and a rather short half-life [51,554]. 

To overcome these challenges, research teams have devised methods to develop neurosteroid analogs possessing more advantageous physicochemical properties, thus broadening the scope through structure–activity relationship investigations. Such efforts have culminated in the synthesis of more favorable allopregnanolone analogs, including the FDA-approved zuranolone and ganaxolone, for the treatment of postpartum depression and seizures, respectively. Zuranolone was identified through C-21 modifications of 5β-nor-19-pregnan-20-one, while ganaxolone was derived from the addition of a methyl substitution in the β-orientation, aimed at mitigating the rapid oxidation of the 3α-hydroxy group of allopregnanolone [555,556,557].

A library of pregnenolone derivatives was synthesized and evaluated in cell cultures to identify compounds that resisted metabolism into other steroids by endogenous enzymes. AEF0117 [3β-(4-methoxybenzyloxy)pregn-5-en-20-one] stood out among these derivatives and displayed optimal pharmacokinetic characteristics, brain penetrance, selectivity, and safety profile, positioning it as a promising candidate for therapeutic intervention targeting the endocannabinoid system [66].

With these three exemplary instances leading the charge in synthetic pregnane class neurosteroid-based therapies, the realm of potential future discoveries remains promising.

Integrating chemical biology for clickable and photoactivatable neurosteroid probe synthesis, along with chemoproteomics to map neurosteroid-protein interactions in live cells, is crucial for advancing targeted therapies. By employing clickable and photoactivatable probes, researchers can selectively label neurosteroid-binding proteins within live cells, allowing for precise mapping of these interactions. Furthermore, by integrating chemoproteomics, researchers can comprehensively profile neurosteroid-protein interactions across different cell types, providing a deeper understanding of the cellular response to neurosteroids [303,304]. However, there is a limitation to using such modified neurosteroids with linkers, as they may reduce neurosteroid potency. Endogenous neuroactive steroid 3α,5α-THDOC and synthetic neuroactive steroid SGE-516 (Sage Therapeutics, Inc., Cambridge, MA, USA) [558] exhibited lower effectiveness in inhibiting TLR4 and TLR7 pathway activation in macrophages from female donors compared to allopregnanolone. This discrepancy in effectiveness is influenced by structural variances, particularly in the C-21 position with hydroxyl or triazole groups [37].

The sex-specific effects of pregnane neurosteroids, particularly in modulating TLR pathway signaling, are of significant interest due to their dual roles in inhibiting inflammatory responses and enhancing anti-inflammatory responses. Understanding the structural requirements for these effects is crucial for developing targeted therapies. 3α,5α-THDOC and SGE-516 inhibits TLR4 and TLR7 pathway activation in macrophages from female donors but not from male donors [37]. Additionally, allopregnanolone has been found to enhance anti-inflammatory TLR4 signaling and increase IL-10 levels in male P rat brains but not female P rat brains [80]. These findings highlight the need for further research to identify other natural and synthetic compounds that mimic the anti-inflammatory activities of endogenous pregnane neurosteroids. Developing compounds with similar properties could lead to targeted therapies for inflammatory conditions, considering sex-specific responses and structural requirements for optimal efficacy.

In summary, the field of neurosteroid treatment research presents a promising avenue for addressing the intricate mechanisms involved in neuropsychiatric diseases. By combining traditional techniques with advanced methodologies such as chemical biology and chemoproteomics, researchers can gain valuable insights into neurosteroid–protein interactions within live cells. Furthermore, the sex-specific effects of pregnane neurosteroids on inflammatory pathways highlight the importance of considering biological variability in therapeutic development. Despite these challenges, continued exploration of neurosteroids holds great promise for advancing our understanding of neuropsychiatric disorders and developing effective treatment strategies tailored to individual patient needs. 

## 6. Conclusions

Neurosteroid therapy shows promise in modulating neuroimmune and immune responses in neurological and psychiatric disorders. Notably, pregnane neuroactive steroids like allopregnanolone and pregnenolone exhibit potent anti-inflammatory effects by influencing TLR activation and its associated signaling pathways. These steroids effectively suppress pro-inflammatory MyD88-dependent TLR signal activation through specific mechanisms, disrupting essential protein–protein interactions necessary for TLR activation and decreasing the production of pro-inflammatory factors. Specifically, they block the binding of TLR2, TLR4, and TLR7 with MyD88, as well as the binding of TLR4 with MD2 and the α2 subunit of GABA_A_ receptors. This inhibition prevents the initiation of inflammatory TLR pathways, resulting in reduced levels of TRAF6 and diminished activation of TAK1, NF-κB, and MAPK/ERK1/2. Consequently, this inhibits the activation of various transcription factors, including CREB, STAT1, ATF2, and IRF7, leading to a decrease in inflammatory mediators such as MCP-1, TNF-α, IL-6, IL-1β, and HMGB1. Moreover, pregnane neuroactive steroids may attenuate inflammatory TLR signaling by promoting the ubiquitination and degradation of TLRs and TLR adapter proteins like TIRAP, as well as by modulating CB1 receptors that potentially interact with TLRs.

Additionally, progesterone suppresses pro-inflammatory TLR activation by increasing IκBα expression, thereby reducing NF-κB phosphorylation and nuclear translocation. It also diminishes the expression of TLRs, MyD88, and CD14, resulting in decreased production of inflammatory cytokines. Direct effects of progesterone on TLR activation have not yet been studied, but it is highly likely that inhibition could be observed, based on the structural similarities to pregnenolone and allopregnanolone at the C and D rings. Further studies are warranted to address this possibility.

Conversely, allopregnanolone enhances anti-inflammatory TRIF-dependent TLR4 signaling in a sex-specific manner, resulting in increased production of anti-inflammatory cytokines and neurotrophic factors such as IL-10 and BDNF. This intricate process involves several steps: allopregnanolone triggers the translocation of TLR4 from the plasma membrane to endosomes by upregulating the p110δ isoform of PI3K and degrading TIRAP. Consequently, there is an accumulation of TLR4 and TRIF, forming a complex within endosomes, thereby activating the anti-inflammatory signaling cascade. Furthermore, allopregnanolone enhances this pathway by increasing activated TRAM levels, leading to upregulation of the transcription factor SP1 and subsequent IL-10 production. Additionally, allopregnanolone elevates BDNF levels, potentially amplifying IL-10 production and release. Moreover, allopregnanolone stimulates the accumulation of endosomal Ras-related protein Rab7, suggesting a role in influencing the equilibrium between pro-inflammatory and anti-inflammatory TLR4 signaling pathways.

The inhibition of inflammatory TLR pathways and enhancement of anti-inflammatory TLR pathways by allopregnanolone have been observed in the brains of alcohol-preferring rats with innately activated TLR pathways, as well as in mouse and human macrophages and mouse microglial cells activated by TLR ligands. 

Of significance is the demonstrated inhibition of inflammatory TLR4 and TLR7 pathways by allopregnanolone (brexanolone) in whole blood cells obtained from individuals with PPD. These were accompanied by symptom improvements. Specifically, brexanolone infusion reduced whole blood cell TNF-α and IL-6, and these effects were correlated with HAM-D score improvement. Furthermore, brexanolone infusion prevented LPS- and imiquimod-induced elevation of TNF-α, IL-1β, and IL-6 in vitro in PPD whole blood cells, indicating inhibition of TLR4 and TLR7 responses. Finally, inhibition of TNF-α, IL-1β, and IL-6 responses to both LPS and imiquimod were correlated with HAM-D score improvements (see Table 1).

Androstane neuroactive steroids, including estrogens, androgens, DHEA, and ADIOL, exert diverse effects on TLR expression and activation, contingent upon the context and specific diseases. ADIOL shows promise as a potent anti-inflammatory agent, while estrogens and androgens exhibit both anti-inflammatory and pro-inflammatory effects on TLRs. DHEA modulates immune responses via TLR activation, although its effectiveness in certain models remains limited and its impact on TLR expression yields complex outcomes.

Under various neuropsychiatric conditions, neuroimmune signaling, involving intricate communication between neuronal and glial cells, becomes disrupted to varying extents, depending on the severity of the disease. This disruption often results in the upregulation of inflammatory TLR ligands and the overactivation of inflammatory TLR signaling pathways, leading to an imbalance in pro-inflammatory and anti-inflammatory factors, favoring the former. Neuroactive steroids like allopregnanolone possess the ability to inhibit inflammatory TLR pathways and promote anti-inflammatory ones. This capability may help maintain homeostatic balance between inflammatory and anti-inflammatory factors in both the brain and periphery.

In numerous neuropsychiatric disorders, there’s an observed overactivation of inflammatory TLR pathways alongside reduced levels of endogenous neurosteroids. While direct studies demonstrating neurosteroid modulation of TLR pathways are primarily seen in conditions like PPD, it is reasonable to hypothesize that administering neurosteroids could, to some extent, improve neuropsychiatric outcomes by modulating TLR activity across various neuropsychiatric disorders (see Table 1). Further research in this direction is imperative for a comprehensive understanding and the development of potential therapeutic strategies.

## 7. Patents

ALM and IB hold a provisional patent on the anti-inflammatory effects of pregnane neurosteroids.

## Figures and Tables

**Table 1:** Effects of neurosteroids on neuropsychiatric disease outcomes. Abbreviations: 3α,5α-THDOC: 3α,5α-tetrahydrodeoxycorticosterone; 3α,5β-THP: (3α,5β)3-hydroxypregnan-20-one or 3α,5β-tetrahydroprogesterone or pregnanolone; ACTH: adrenocorticotropic hormone; AD: Alzheimer’s disease; ADAS-Cog: Alzheimer’s Disease Assessment Scale–Cognitive subscale; AEA: anandamide; AEF0117: 3β-(4-methoxybenzyloxy)pregn-5-en-20-one; AEs: adverse events; ASD: autism spectrum disorder; AUD: alcohol use disorder; BDI: Beck Depression Inventory; BPD: bipolar depression; BRMS: Bech–Rafaelsen Melancholia Scale; CDD: cyclin-dependent kinase-like 5; CGI-I: Clinical Global Impression–Improvement; CUD: cocaine use disorder ; DES: Differential Emotion Scale; DHEA: dehydroepiandrosterone; DHEAS: dehydroepiandrosterone sulfate; EPDS: Edinburgh Postnatal Depression Scale; GAD-7: Generalized Anxiety Disorder 7-item Scale; GDS: Geriatric Depression Scale; HAD-D: Hospital Anxiety and Depression Scale–Depression; HRSD & HAM-D: Hamilton Rating Scale for Depression; HRSA: Hamilton Rating Scale for Anxiety; IDS-SR: Inventory of Depressive Symptomatology-Self Report; IL: interleukin; LPS: lipopolysaccharide; MADRS: Montgomery-Åsberg Depression Rating Scale; MoCA: Montreal Cognitive Assessment Test; MRI: magnetic resonance imaging; PHQ-9: Patient Health Questionnaire; RAVLT: Rey Auditory Verbal Learning Test; SAEs: serious adverse events; TLR: toll-like receptor; TMT: Trail Making Test-B; TNF: tumor necrosis factor; VAS: 10-point Visual Analog Scale; YMRS: Young Mania Rating Scale; y.o.: years old.

Disease	Neurosteroid /Dose/Route of Administration	Subjects/Age/Sex	Outcomes	References
Postpartum Depression	Brexanolone 5 mg/mL in 250 mg/mL sulfobutylether-β-cyclo-dextrin, 60 hs continuous intravenous infusion (30 μg/kg per h (0–4 h); 60 μg/kg per h (4–24 h); 90 μg/kg per h (24–52 h); 60 μg/kg per h (52–56 h); 30 μg/kg per h (56–60 h).)	Women with PPD (n = 18), 24–41 years old (y.o.)	Brexanolone infusion reduced whole blood cell TNF-α and IL-6 and these effects were correlated with HAM-D score improvement. Brexanolone infusion prevented LPS- and imiquimod-induced elevation of TNF-α, IL-1β and IL-6 in whole blood cells in vitro, indicating inhibition of TLR4 and TLR7 responses. Inhibition of TNF-α, IL-1β and IL-6 responses to both LPS and imiquimod were correlated with HAM-D score improvements. Brexanolone infusion led to significant increases in allopregnanolone and 3α,5α-THDOC levels, while causing decreases in 3α,5α-androsterone and 3α,5α-androstandiol levels. Pregnenolone and 3α,5β-THP levels remained unchanged following infusion. However, there was no observed correlation between the percentage change in steroid levels post-infusion and the improvement in HAM-D scores.	Balan et al., 2023 [64]
Postpartum Depression	Brexanolone 5 mg/mL in 250 mg/mL sulfobutylether-β-cyclo-dextrin, 60 h continuous intravenous infusion (30 μg/kg per h (0–4 h); 60 μg/kg per h (4–24 h); 90 μg/kg per h (24–52 h); 60 μg/kg per h (52–56 h); 30 μg/kg per h (56–60 h).	Women with PPD (n = 21), 18–45 y.o.	Brexanolone was generally well tolerated in PPD patients. Improvement in depressive symptoms (HAM-D and MADRS scores) was observed up to 30 days after brexanolone treatment.	Kanes et al., 2017 [63]
Postpartum Depression	Brexanolone 5 mg/mL in 250 mg/mL sulfobutylether-β-cyclo-dextrin, 60 h continuous intravenous infusion (30 μg/kg per h (0–4 h); 60 μg/kg per h (4–24 h); 90 μg/kg per h (24–52 h); 60 μg/kg per h (52–56 h); 30 μg/kg per h (56–60 h).	Women with PPD (Study 1 n = 120; Study 2 n = 100), 18–45 y.o.	Brexanolone was generally well tolerated in PPD patients. Common adverse effects included somnolence, dizziness and sedation in 30% of patients. Improvement in depressive symptoms (HAM-D total scores, HAM-D remission and CGI-I response) was observed up to 30 days after brexanolone treatment. No changes in GAD-7, EPDS or PHQ scores were noted.	Meltzer-Brody et al., 2018 [62]
Postpartum Depression	Brexanolone 5 mg/mL in 250 mg/mL sulfobutylether-β-cyclo-dextrin, 60 h continuous intravenous infusion (30 μg/kg per h (0–4 h); 60 μg/kg per h (4–24 h); 90 μg/kg per h (24–52 h); 60 μg/kg per h (52–56 h); 30 μg/kg per h (56–60 h).	Women with PPD (n = 16), 18–45 y.o.	Brexanolone was well tolerated in PPD patients. Improvement in depressive symptoms (HAM-D scores) was observed up to 16 months after brexanolone treatment.	Patterson et al., 2022 [65]
Postpartum Depression	Zuranolone 30 mg, administered orally each evening for 2 weeks.	Women with PPD (n = 150), 18–45 y.o.	Zuranolone was well tolerated in PPD patients; however (one patient experienced a serious adverse effect (confusional state) and abandoned the trial. Improvement in depressive symptoms (HAM-D, MADRS and HRSA scores) up to 45 days after Zuranolone treatment.	Deligiannidis et al., 2021 [359]
Major Depressive Disorder	Zuranolone 30 mg, administered orally each evening for 2 weeks.	Men and women with MDD (n = 89), 18–65 y.o.	Zuranolone was well tolerated in MDD patients. Improvement in depressive symptoms (HAM-D and CGI-I scores) at day 15.	Gunduz-Bruce et al., 2019 [360]
Major Depressive Disorder	Testosterone 50–1000 mg/day, administered for 4–144 weeks	Men with MDD (n = 1890), 27–80 y.o.	Testosterone was well tolerated in patients with MDD. Reduction in depressive symptoms (HDRS, BDI-I/BDI-II, MADRS, PHQ-9, GDS, BRMS or/and HADS-D scores) was observed.	Walther et al., 2019 (Meta-analysis) [362]
Bipolar Depression	Pregnenolone titrated to 500 mg/day, oral administration (100 mg/day for 1 week, 150 mg/day for 3 weeks, 500 mg/day for 8 weeks).	Men and women with BPD (n = 80), 18–75 y.o.	Pregnanolone was well tolerated in BPD patients. Improvement in depressive symptoms (HRSD scores) was observed, but not in anxiety symptoms (HRSA scores) or manic behavior (YMRS scores). A significant depression remission rate in the IDS-SR.	Brown et al., 2014 [43]
Unipolar and Bipolar Depression	Pregnenolone titrated to 100 mg/day for 8 weeks.	Men and women with BPD I or II with episodes of MDD and history of SUD (n = 70), 17–70 y.o.	Pregnenolone was well tolerated in BPD patients. A trend toward significance favoring pregnanolone was detected in depressive (HRSD scores), and manic symptoms (YMRS scores) at the end of the treatment (week 8). No significant effect was observed on the cognitive assessment (RAVLT scores, TMT and Stroop Test).	Osuji et al., 2010 [45]
Cocaine Use Disorder	Progesterone 400 mg/day, oral administration for 7 days.	Men and women with CUD (n = 46), 35–50 y.o.	Progesterone was found to be safe and well tolerated in CUD patients. Increases in allopregnanolone and pregnanolone plasma levels were observed in all patients. No effects on the levels of pregnenolone, testosterone, androstanediol, or DHEA; however, testosterone and androstanediol levels were higher in men than in women.	Milivojevic et al., 2019 [59]
Cocaine Use Disorder	AEF0117, an unmetabolized derivative of pregnenolone: 0.06 mg/day (Cohort I), 1 mg/day (Cohort II), oral administration for 6 days.	Men and women with CUD (n = 29), 21–60 y.o.	AEF0117 (1 mg/day) reduced the subjective effects of cannabis, as measured by the ‘Intoxication’ subscale and the ‘Felt Good Cannabis Effect’ item. AEF0117 also decreased cannabis self-administration, with the 1 mg/day dose showing a greater effect compared to the 0.06 mg/day dose. The sequence of AEF0117 administration impacted outcomes, with AEF0117 maintaining its effects even after a washout period of ≥14 days, likely due to its long elimination half-life. AEF0117 was safe and well tolerated, with no significant treatment-related SAEs. Any observed AEs were similar between the AEF0117 and placebo groups, except for one unrelated severe AE. There was no evidence to suggest that AEF0117 precipitated symptoms of cannabis withdrawal. AEF0117 did not produce significant changes in endocannabinoid levels compared to placebo, except for a slight increase in AEA levels with the lower dose (0.06 mg/day), which was likely not caused by AEF0117 administration. There were minor effects on certain mood ratings, but these did not seem to be clinically relevant and were likely influenced by individual sensitivity to AEF0117’s effects.	Haney et al., 2023 [66]
Alcohol Use Disorder	Pregnenolone 300 mg/day or 500 mg/day, oral administration for 8 weeks.	Men and women with AUD (n = 43), 18–65 y.o.	Pregnenolone was well tolerated in AUD patients. Decreases in alcohol craving scores (stress- and cue-induced). Decrease in anxiety response to stress (VAS scores) in the 300 mg dose group. Normalization of the ACTH/cortisol ratio following stress or alcohol cue was observed at both doses. Normalization of heart rate (stress-induced), systolic blood pressure (stress- and cue-induced), and diastolic blood pressure was noted (cue-induced, only in the 300 mg dose group).	Milivojevic et al., 2023 [61]
Alzheimer’s disease	DHEA (50 mg per os) administered orally 2x/day for 6 months.	Men and postmenopausal women with AD ≥55 y.o.	Increased DHEA and DHEAS levels in serum. No improvements in cognitive performance (ADAS-Cog scores).	Wolkowitz et al., 2003 [488]
Alzheimer’s disease	Intravenous allopregnanolone 1x/week for 12 weeks (doses: 0.3–3 mL).	Men and women with AD or probable AD ≥55 y.o.	Allopregnanolone was safe and well tolerated in all participants, with no significant differences in adverse events between treatment arms. Allopregnanolone levels in plasma reached a T_max_ at 30 min post-infusion (2, 4, and 6 mg doses) and returned to the lower limit of quantification 4 h post-infusion. No indicators of sedation were observed at lower doses of allopregnanolone (2 mg and 4 mg), but increased sedation was observed at higher doses (6–18 mg). After 12 weeks of treatment, there were no statistically significant differences among cohorts in cognitive assessments (ADAS-Cog score, MoCA total score, and Cogstate Brief Battery composite score). MRI imaging data showed no adverse outcomes of allopregnanolone treatment on hippocampal volume, with a trend suggesting decreased atrophy in allopregnanolone-treated participants.	Hernandez et al., 2020 [482]
Pain	Pregnenolone titrated up to 500 mg/day, oral administration (0 mg/day for 1 week, 50 mg twice a day for 1 week, 150 mg twice a day for 1 week, 250 mg twice a day for 2 weeks).	Iraq- and Afghanistan-era male veterans 18–65 years old with chronic lower back pain.	Pregnenolone was well tolerated, with minor adverse effects in subjects. Decreased lower back pain intensity and interference scores following 4 weeks of treatments. Increased levels of pregnenolone and allopregnanolone in serum.	Naylor et al., 2020 [417]
Autism Spectrum Disorder	Pregnenolone Titrated by weight: 20 to 45 kg, up to 2.5 mg/day (0.5 mg/day before sleep for 1 week. 0.5 mg weekly increases were used until 1 mg in the morning and 1.5 mg before sleep). >45 kg, up to 3.5 mg/day. A group received 100 mg twice a day.	36 males and 23 females (11–17 y.o.).	Pregnenolone improved irritability, stereotypy, and hyperactivity in adolescents with ASD. No significant adverse effects were observed when comparing the different treatment groups to the placebo control.	Ayatollahi et al., 2020 [502]
Epilepsy	Ganaxolone 33–63 mg/kg or 900–1800 mg three times a day, oral suspension.	88 patients (79.5% female) 2–19 y.o. with CDD.	Ganaxolone treatments were safe and well tolerated with mild adverse effects. Following 2 years of treatment, motor seizure intensity, duration, and frequency decreased.	Olson et al., 2024 [433]
Epilepsy	40 mg progesterone daily in second half of the cycle from 15th to 25th day, oral administration.	38 patients 18–45 y.o. women with catamenial epilepsy.	Treatments involving progesterone have shown promise in reducing seizure frequency in women with intractable catamenial epilepsy.	Najafi et al., 2013 [426]

## Abbreviations

5α-DHDOC/5β-DHDOC: 5α- or 5β-dihydrodeoxycorticosterone; 5α-DHP/5β-DHP: 5α- or 5β-dihydroprogesterone; 3α,5α-THDOC: 3α,5α-tetrahydrodeoxycorticosterone or [3α,5α]-3,21-dihydroxypregnan-20-one; 3α,5α-THP: (3α,5α)3-hydroxypregnan-20-one or 3α,5α-tetrahydroprogesterone or allopregnanolone; 3α,5β-THP: (3α,5β)3-hydroxypregnan-20-one or 3α,5β-tetrahydroprogesterone or pregnanolone; 3α-HSD/3β-HSD: 3α- or 3β-hydroxysteroid dehydrogenase; 3α-diol: androstanediol; 3α,5β-PC: 3α,5β-20-oxo-pregnane-3-carboxylic acid; 3β,5β-THP: Epiallopregnanolone; 17β-HSD: 17β-hydroxysteroid dehydrogenases; 6-OHDA: 6-hydroxydopamine; AD: Alzheimer’s disease; ADIOL: androstenediol or 5-androstenediol or 5α-androstane-3β,17β-diol; AEF0117: 3β-(4-methoxybenzyloxy)pregn-5-en-20-one; Akt: protein kinase B; AMPA: α-amino-3-hydroxy-5-methyl-4-isoxazolepropionic acid; AR: Androgen receptors; ASD: Autism spectrum disorder; ATF2: Activating transcription factor 2; AUD: Alcohol use disorder; BDNF: Brain-derived neurotrophic factor; BPD: Bipolar disorder; CB1: cannabinoid receptor type 1; CD14: Cluster of differentiation 14 protein; CDD: cyclin-dependent kinase-like 5; CNS: Central nervous system; COX-2: Cyclooxygenase-2; CREB: Cyclic adenosine monophosphate response element-binding protein; CRF: Corticotropin-releasing factor; CSF: Cerebrospinal fluid; CYP11A1: Cytochrome P450scc or cholesterol side-chain cleavage enzyme; CYP21A2: Cytochrome P450 21A2 or 21-hydroxylase; CUD: Cocaine use disorder; CX3CL1: C-X3-C motif chemokine ligand 1 (Fractalkine); DHEA: Dehydroepiandrosterone; DHEAS: Dehydroepiandrosterone sulfate; DHT: Dihydrotestosterone; DOC: Deoxycorticosterone; EPS: exopolysaccharide; ER: Estrogen receptor; ERK: Extracellular signal-regulated kinase; FDA: Food and Drug Administration; GABA_A_ receptor_:_ Gamma-aminobutyric acid receptor type A; GB: Glioblastoma; GFAP: Glial fibrillary acidic protein; HAM-D: Hamilton Rating Scale for Depression; HMGB1: High mobility group box 1; HPA: Hypothalamic-pituitary-adrenal; IFN: interferon; IκB: Inhibitor of kappa-B; IKK: IκB kinase; IL: Interleukin; IL-1R: Interleukin-1 receptor; iNOS: inducible nitric oxide synthase; IRAK: Interleukin-1 receptor-associated kinase; IRF: IFN regulatory factor; JNK: c-Jun N-terminal kinase; LPS: Lipopolysaccharide; MAP2: Microtubule-associated protein 2; MAPKs: Mitogen-activated protein kinases; MCP-1: Monocyte chemotactic protein-1; MD2: Myeloid differentiation protein 2; MDD: Major depressive disorder; mPRs: Membrane progesterone receptors; MS: Multiple Sclerosis; MyD88: Myeloid differentiation primary response 88; NF-κB: Nuclear factor kappa-B; NMDA: N-methyl-D-aspartate; NO: Nitric oxide; p: phosphorylated; P: alcohol-preferring; PBMC: Peripheral blood mononuclear cells; PD: Parkinson’s disease; PI3K: Phosphoinositide 3-kinase; PPD: Postpartum depression; PS: Pregnenolone sulfate; PtdIns(4,5)P2: Phosphatidylinositol-(4,5)-bisphosphate; PTSD: Post-traumatic stress disorder; Rab7: Ras-related protein Rab7; S100β: S100 calcium-binding protein B; SOCS: Suppressor of cytokine signaling; SP1: Specificity protein 1; STAT1: Signal transducer and activator of transcription 1; TAK1: Transforming growth factor beta-activated kinase 1; TBI: Traumatic brain injury; TGF: Transforming growth factor; TH: Tyrosine hydroxylase; THC: Δ9-tetrahydrocannabinol; THDOC: Tetrahydrodeoxycorticosterone ([3α,5α]-3,21-dihydroxypregnan-20-one 3α,5α-THDOC); TIRAP: Toll/interleukin-1 receptor domain-containing adapter protein; TLR: Toll-like receptor; TNF: Tumor necrosis factor; TRAF: TNF receptor-associated factor; TRAM: TRIF-related adapter molecule; TRIF: IFN-β toll/interleukin-1 receptor domain-containing adapter-inducing.
